# Supplementary material for: Anisotropic charge trapping in phototransistors unlocks ultrasensitive polarimetry for bionic navigation
Source: Nat Commun. 2022 Nov 4;13:6629. doi: 10.1038/s41467-022-34421-3 (PMC9636252; doi:10.1038/s41467-022-34421-3)
Supplement: Supplementary file 1 — Supplementary Information [file 41467_2022_34421_MOESM1_ESM.pdf]

Supplementary Information

# Anisotropic charge trapping in phototransistors unlocks ultrasensitive polarimetry for bionic navigation

**Jing Pan[1#], Yiming Wu[2#], Xiujuan Zhang[1]*, Jinhui Chen[1], Jinwen Wang[1], Shuiling Cheng[1], Xiaofeng Wu[1], Xiaohong Zhang[1]*, and Jiansheng Jie[1,3]***

[1]Institute of Functional Nano & Soft Materials (FUNSOM), Jiangsu Key Laboratory for Carbon-Based Functional Materials & Devices, Soochow University, Suzhou, Jiangsu 215123, P. R. China

[2]Institute of Materials Research and Engineering, Agency for Science, Technology and Research (A*STAR), Singapore 138634, Singapore

[3]Macao Institute of Materials Science and Engineering, Macau University of Science and Technology, Taipa, Macau SAR 999078, P. R. China

[#]These authors contributed equally: Jing Pan, Yiming Wu

*E-mail: xjzhang@suda.edu.cn, xiaohong_zhang@suda.edu.cn, jsjie@suda.edu.cn

**Table of Contents**

I. Compact model of organic field-effect transistors....2

II. Theoretical estimation of the anisotropic charge trapping effect....6

III. Fabrication and characterizations of C8-BTBT crystal array....13

IV. Optical anisotropy analyses of C8-BTBT....20

V. Calculations of the device performance....25

VI. Characterizations of interfacial trap sites on $SiO_2$....30

VII. Analyses of polarization-dependent photoresponse....36

VIII. Theoretical estimation of partially polarized light detection....53

IX. Polarization navigation measurements....55

X. Supplementary References....62

## I. Compact model of organic field-effect transistors

We utilized the compact model of organic field-effect transistors (OFETs) developed by Estrada *et al.*[1,2] to simulate the transfer characteristics of a phototransistor. The model can well explain the drain current ($I_{DS}$) both in the above-threshold regime and the sub-threshold regime. The above-threshold drain current ($I_{above}$) of a p-type OFET can be described as the product of three parts: channel conductance ($g_{ch}$), effective drain voltage ($V_{DSe}$) for a smooth linear-to-saturation transition, and a modified asymptotic expression $[1+\lambda_s(|V_{DS}|-\alpha_s|V_G-V_{th}|)]$ for better describing the output conductance:

$$\begin{aligned} I_{above} &= g_{ch}V_{DSe}[1+\lambda_s(|V_{DS}|-\alpha_s|V_G-V_{th}|)] \\ &= -\frac{\frac{W}{L}C_i\mu_{FET}(V_G-V_{th})}{1-R_c[\frac{W}{L}C_i\mu_{FET}(V_G-V_{th})]}\cdot\frac{V_{DS}[1+\lambda_s(|V_{DS}|-\alpha_s|V_G-V_{th}|)]}{\left[1+\left|V_{DS}/[\alpha_s(V_G-V_{th})]\right|^m\right]^{1/m}} \end{aligned} \tag{S1}$$

where $W$ and $L$ are the channel width and length of the device, respectively, $C_i$ is the unit-area capacitance of the dielectric layer, $\mu_{FET}$ is the field-effect mobility, $V_G$ is the gate voltage, $V_{th}$ is the threshold voltage, $V_{DS}$ is the drain voltage, $R_c$ is the contact resistance, $m$ is the linear-to-saturation transition parameter, $\lambda_s$ is the saturation coefficient, and $\alpha_s$ is the saturation modulation parameter. For simplicity, we now consider an ideal case where $R_c = 0$, and a $V_G$-independent intrinsic mobility of the active material ($\mu_{in}$) is used instead of $\mu_{FET}$. $I_{above}$ can thus be written as:

$$I_{above} \approx \frac{-\frac{W}{L}C_i\mu_{in}(V_G-V_{th})V_{DS}[1+\lambda_s(|V_{DS}|-\alpha_s|V_G-V_{th}|)]}{\left[1+\left|V_{DS}/[\alpha_s(V_G-V_{th})]\right|^m\right]^{1/m}} \tag{S2}$$

The sub-threshold drain current ($I_{sub}$) can be expressed as:

$$I_{sub} = I_0\exp\left[-\frac{\ln 10}{\mathrm{SS}}(V_G-V_{on})\right] \tag{S3}$$

where $I_0$ is the off-state current, SS is the subthreshold swing, and $V_{on}$ is the onset voltage. A hyperbolic tangent transition function is used to combine the two regimes together. The total $I_{DS}$ is:

$$I_{total} = I_{above}\cdot\frac{1}{2}[1-\tanh[B(V_G-V_B)]]+I_{sub}\cdot\frac{1}{2}[1+\tanh[B(V_G-V_B)]]+I_0 \tag{S4}$$

Note that here two additional parameters, namely transition voltage ($V_B$) and transition parameter ($B$) are used for smooth transition between $I_{above}$ and $I_{sub}$. Because $I_{above}$ exhibits a steep drop when approaching $V_{th}$, $V_B$ is normally chosen to be a few volts away from $V_{th}$ toward the above-threshold

direction for a better transition. Supplementary Table 1 lists the values of the parameters used for describing an ideal OFET, note that the values of $W$, $L$, $C_i$ are based on the device structure of our organic phototransistor (OPT), and other values were set carefully to ensure a smooth and ideal transfer curve[1] (Supplementary Fig. 1).

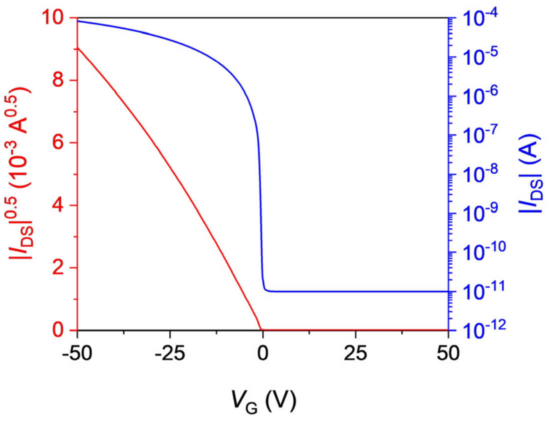


**Supplementary Figure 1 | Model construction of an ideal OFET.** Transfer characteristics of an ideal OFET described by the compact model.

**Supplementary Table 1** | Parameter settings for describing an ideal OFET.

| Parameter | Value | Note |
|---|---|---|
| $W$ | 75 μm | Channel width |
| $L$ | 25 μm | Channel length |
| $C_i$ | $1.8\times10^{-8}$ F cm$^{-2}$ | Capacitance of a 200 nm-thick $SiO_2$ layer |
| $\mu_{in}$ | 1 cm$^{2}$ V$^{-1}$ s$^{-1}$ | Intrinsic mobility |
| $V_{th}$ | 0 V | Threshold voltage |
| $V_{on}$ | 0 V | Onset voltage |
| $\lambda_s$ | $-1\times10^{-3}$ V$^{-1}$ | Saturation coefficient |
| $\alpha_s$ | 1 | Saturation modulation parameter |
| $m$ | 1.8 | Linear-to-saturation transition parameter |
| SS | 1 V dec$^{-1}$ | Subthreshold swing |
| $I_0$ | $-1\times10^{-11}$ A | Off-state current |
| $B$ | 3 V$^{-1}$ | Below-to-above-threshold transition parameter |
| $V_B$ | -1 V | Transition voltage |

## II. Theoretical estimation of the anisotropic charge trapping effect

Generally, there are two working modes in a phototransistor, namely the photoconductive (PC) mode and the photovoltaic (PV) mode[3]. The PC mode is related to the change of the channel conductance due to the difference in charge carrier concentration upon light illumination. Therefore, in an ideal PC mode where photogenerated electrons and holes are effectively separated under the electric field, and are totally collected by the electrodes before recombination, the photocurrent ($I_{ph}$) increases linearly with the amount of light absorbed. Therefore, assuming the device is illuminated by a parallelly ( $\parallel$ ) or perpendicularly ( $\perp$ ) polarized light with the electric field $\mathbf{E}_{\parallel}$ or $\mathbf{E}_{\perp}$ orienting to the strongest or weakest light absorption direction, respectively, the anisotropic ratio of optical absorbance ($a_{in}$) should determine the upper limit of the dichroic ratio (DR) of the channel current (Supplementary Fig. 2a).

In terms of the PV mode, light absorption will result in the trapping of photogenerated charge carriers and the shift of $V_{th}$ (Supplementary Fig. 2b). For an anisotropic photoactive crystal, the total light power absorbed is determined by the product of optical absorbance and incident light power[4]:

$$P_{abs} = A_{abs} P_{in} \tag{S5}$$

Therefore, the effect of polarization-dependent light absorption can be analogous to the result of changing light power of an unpolarized light (*i.e.*, assigning $a_{in}P_{eff}$ and $P_{eff}$ to represent $P_{abs}$ in the parallel and perpendicular polarization states, respectively). Based on Equation 2 in the main text, we utilized the values listed in Supplementary Table 2 to predict $\Delta V_{th}$ under polarized light (ideally assuming quantum efficiency = 1). The value of the empirical parameter $N$ was selected based on the fitting curve for the experimental data in Supplementary Fig. 16d. Therefore, for $\boldsymbol{E}_{\parallel}$ we can write down:

$$\Delta V_{th,\parallel} = \frac{775.6\times(1.38\times10^{-23}\ \mathrm{JK^{-1}})\times(298.15\,\mathrm{K})}{1.6\times10^{-19}\,\mathrm{C}} \ln\left[1+\frac{(1.6\times10^{-19}\,\mathrm{C})\times(365\times10^{-9}\ \mathrm{m})\times a_{in}P_{eff}}{(5\times10^{-11}\ \mathrm{A})\times(6.626\times10^{-34}\,\mathrm{Js})\times(3\times10^{8}\ \mathrm{ms^{-1}})}\right] \tag{S6}$$

for $\boldsymbol{E}_{\perp}$ we have:

$$\Delta V_{th,\perp} = \frac{775.6\times(1.38\times10^{-23}\ \mathrm{JK^{-1}})\times(298.15\,\mathrm{K})}{1.6\times10^{-19}\,\mathrm{C}} \ln\left[1+\frac{(1.6\times10^{-19}\,\mathrm{C})\times(365\times10^{-9}\ \mathrm{m})\times P_{eff}}{(5\times10^{-11}\ \mathrm{A})\times(6.626\times10^{-34}\,\mathrm{Js})\times(3\times10^{8}\ \mathrm{ms^{-1}})}\right] \tag{S7}$$

The threshold voltage difference between the two polarization states is thus:

$$\Delta V_{\mathrm{th,PD}} = \Delta V_{\mathrm{th},\parallel} - \Delta V_{\mathrm{th},\perp} = 19.94 \times \ln\left(\frac{1+5.88\times10^{9}\times a_{\mathrm{in}}P_{\mathrm{eff}}}{1+5.88\times10^{9}\times P_{\mathrm{eff}}}\right)\ \mathrm{V} \tag{S8}$$

According to the above equation, as $P_{\mathrm{eff}}$ increases, $\Delta V_{\mathrm{th,PD}}$ will gradually become saturated (Supplementary Fig. 3a). Therefore, for a particular $a_{\mathrm{in}}$, we can estimate the upper limit of $\Delta V_{\mathrm{th,PD}}$ that can be obtained. *E.g.*, for C8-BTBT crystals we used for fabricating OPTs in following discussions, the maximum $\Delta V_{\mathrm{th,PD}}$ is estimated to be ~34.2 V with $a_{\mathrm{in}} = 5.6$ (Supplementary Fig. 3b). Since our estimations are based not on the absolute value of $V_{\mathrm{th}}$ but on the change in $V_{\mathrm{th}}$ under polarized light, the exact position of threshold/onset point in the dark does not impact our conclusions. Therefore, we utilize the compact model in Section I and assign the threshold/onset point of $I_{\mathrm{light},\perp}$ as reference zero. The relative value of $I_{\mathrm{light},\parallel}$ can be attained with $\Delta V_{\mathrm{th,PD}} = \Delta V_{\mathrm{on,PD}} = \Delta V_{\mathrm{B,PD}} = 34.2$ V, and further the $a_{\mathrm{in}}$-related DR can be predicted. Note that the subthreshold current $I_0$ may be higher under light illumination due to the generation of excess charge carriers. Therefore, $I_0$ is set to be $10^{-9}$ A for a more accurate prediction in our case. The DR can reach over $10^4$ in PV mode compared to the limited value (DR $\leq$ 5.6) in PC mode (Supplementary Fig. 4a). Further, it is suggested that fabricating high-quality photoactive crystals with a larger $\mu_{\mathrm{in}}$ is favorable for attaining a much higher DR (Supplementary Fig. 4b).

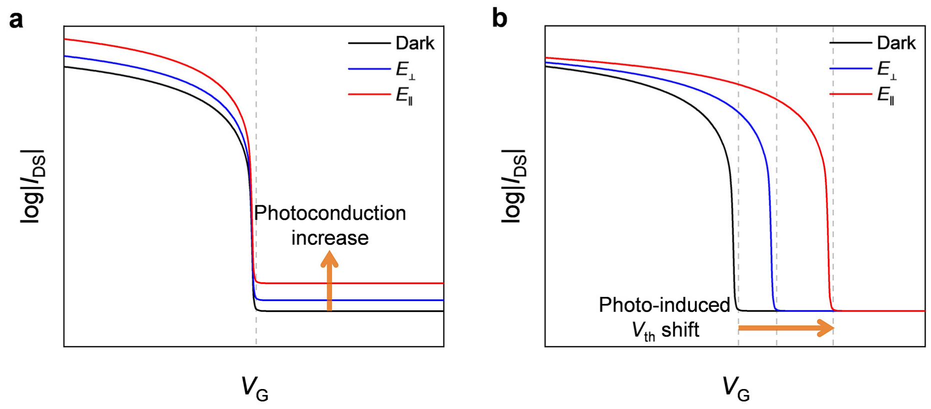


**Supplementary Figure 2 | Different working modes of a phototransistor.** Schematics of polarization-dependent transfer curves of a phototransistor ideally working in **a,** PC mode and **b,** PV mode.

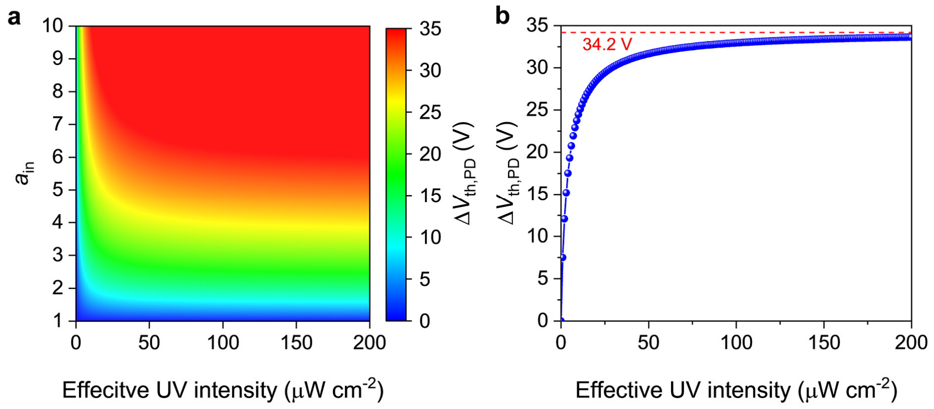


**Supplementary Figure 3 | Estimations of the UV intensity-dependent anisotropic charge trapping effect. a,** Contour plot of predicted $\Delta V_{th,PD}$ versus $a_{in}$ and effective UV intensity. **b,** Predicted relationship between $\Delta V_{th,PD}$ and effective UV intensity with $a_{in} = 5.6$.

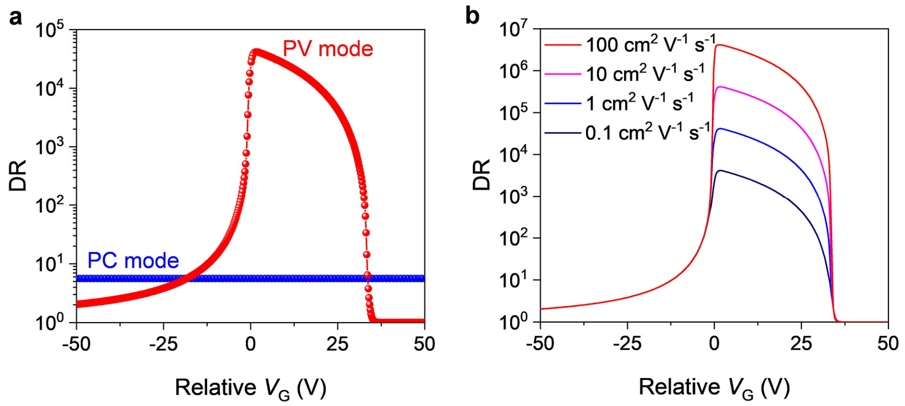


**Supplementary Figure 4 | Estimations of the possible DRs obtained via the anisotropic charge trapping effect. a,** Predicted DRs in PC mode (blue curve) and PV mode (red curve) of a phototransistor with $\mu_{\mathrm{in}}$ = 1 cm$^2$ V$^{-1}$ s$^{-1}$ and $a_{\mathrm{in}}$ = 5.6. **b,** Predicted influence of $\mu_{\mathrm{in}}$ on DR ($a_{\mathrm{in}}$ = 5.6). The relative $V_{\mathrm{G}}$ represents the applied gate voltage relative to the reference onset/threshold point of $I_{\mathrm{light},\perp}$.

**Supplementary Table 2** | Parameter settings for describing $\Delta V_{th}$ of an ideal organic phototransistor under light illumination.

| Parameter | Value | Note |
| --- | --- | --- |
| $W \times L$ | $75 \times 25\ \mu m^2$ | Device area |
| $N$ | 775.6 | Empirical parameter |
| $k$ | $1.38 \times 10^{-23}$ J K$^{-1}$ | Boltzmann constant |
| $T$ | 298.15 K | Room temperature |
| $q$ | $1.6 \times 10^{-19}$ C | Elementary charge |
| $\lambda$ | 365 nm | Incident light wavelength |
| $I_d$ | $5 \times 10^{-11}$ A | Dark current in the off-state |
| $h$ | $6.626 \times 10^{-34}$ J s | Planck's constant |
| $c$ | $3 \times 10^{8}$ m s$^{-1}$ | Speed of light |

**Supplementary Table 3** | Comparison of the C8-BTBT crystal with previously reported anisotropic photoactive semiconductors. $a_{in}$ here refers to the anisotropic ratio of optical absorbance.

| Material | $\lambda$ (nm) | $a_{in}$ | Ref. |
|---|---|---|---|
| C8-BTBT | 365 | 5.6 | This work |
| GeSe | 808 | 3.02 | [5] |
| Te | 2300-3300 | ~10 | [6] |
| $SbI_3 \cdot 3S_8$ | 430 | 3.9 | [7] |
| $CsPbBr_3$ | ~517 | 2.8 | [8] |
| $(BA)_2(MA)Pb_2Br_7$ | 405 | 1.9 | [9] |
| DPA | 436 | 3.97 | [10] |

## III. Fabrication and characterizations of C8-BTBT crystal array

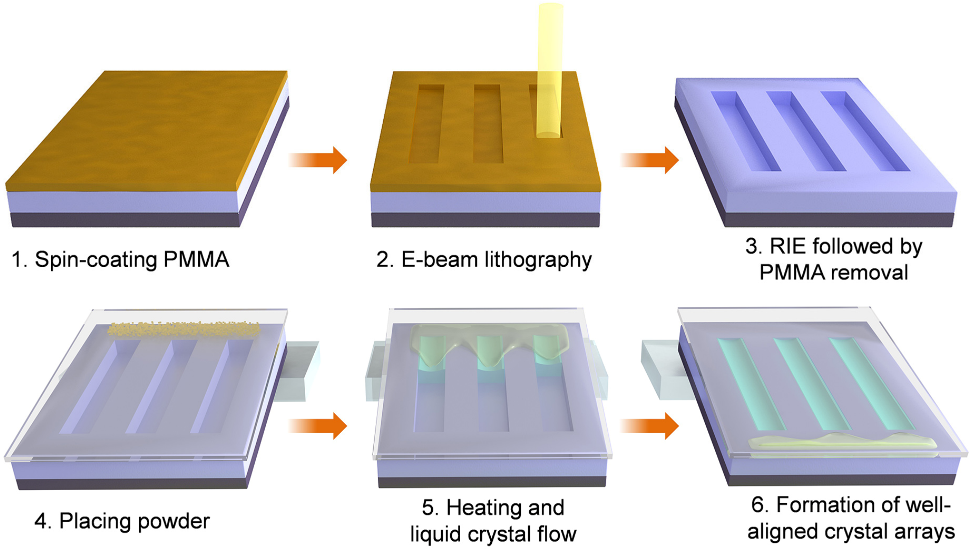


**Supplementary Figure 5 | Fabrication of aligned C8-BTBT crystals.** Schematic illustrations of the fabrication process of the C8-BTBT crystal array.

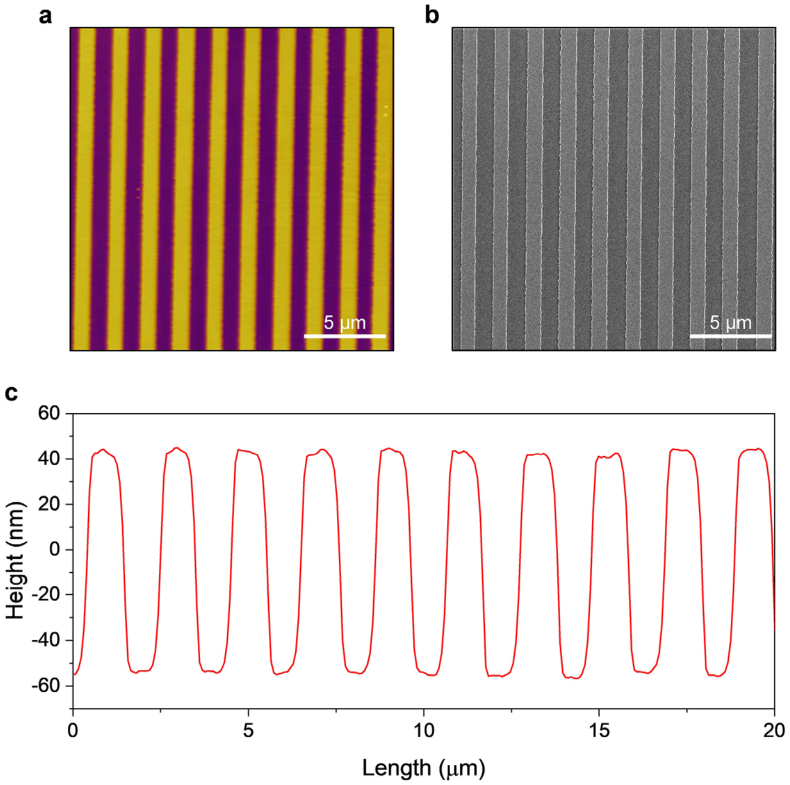


**Supplementary Figure 6 | Morphological characterizations of $SiO_2$ micro-channels. a,** AFM and **b,** SEM characterizations of the periodic $SiO_2$ micro-channels. **c,** Corresponding height profile of the $SiO_2$ micro-channels. The height and width of the channel are ~100 nm and ~1 μm, respectively.

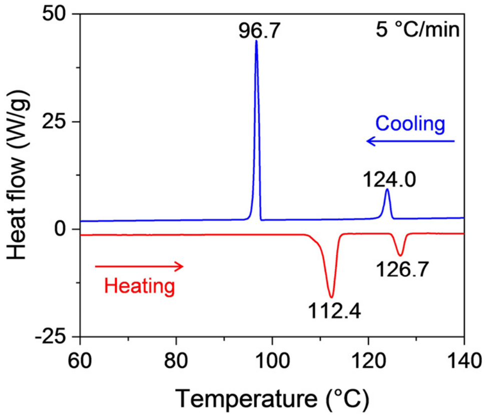


**Supplementary Figure 7 | Determination of the heating temperature of C8-BTBT powder.** Difference scanning calorimeter thermogram of C8-BTBT powder. The heating and cooling speed is set to be 5°C/min. The smectic liquid crystal phase and the isotropic phase of C8-BTBT appears at 112.4°C and 126.7°C upon heating, respectively. Therefore, a heating temperature of 130°C is used to acquire free-flowing C8-BTBT liquid for channel-restricted growth.

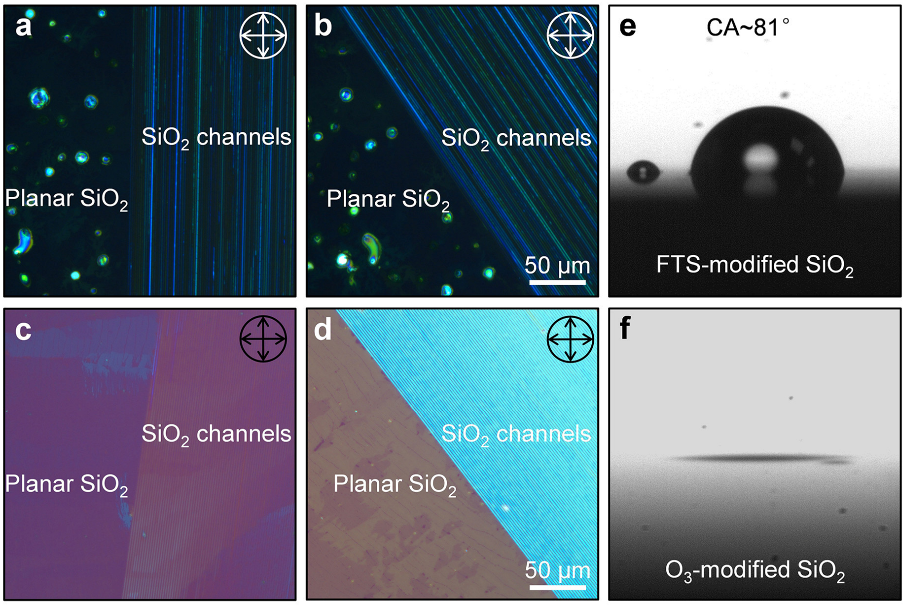


**Supplementary Figure 8 | Comparisons of C8-BTBT arrays fabricated via different surface modifications on $SiO_2$.** CPOM images of C8-BTBT grown on **a and b,** FTS-modified and **c and d,** $O_3$-modified $SiO_2$ templates. Contact angle (CA) measurements of the C8-BTBT liquid droplet on **e,** FTS-modified and **f,** $O_3$-modified $SiO_2$ templates. The surface modification was found to have a large influence on the flow of C8-BTBT liquid. Lyophobic treatment by FTS (CA~81°) results in the pinning of C8-BTBT liquid droplets on $SiO_2$ and differently oriented C8-BTBT polycrystals after cooling, while lyophilic treatment by $O_3$ (negligible CA) can lead to free-flowing C8-BTBT liquid on $SiO_2$ and uniformly oriented C8-BTBT crystals after cooling.

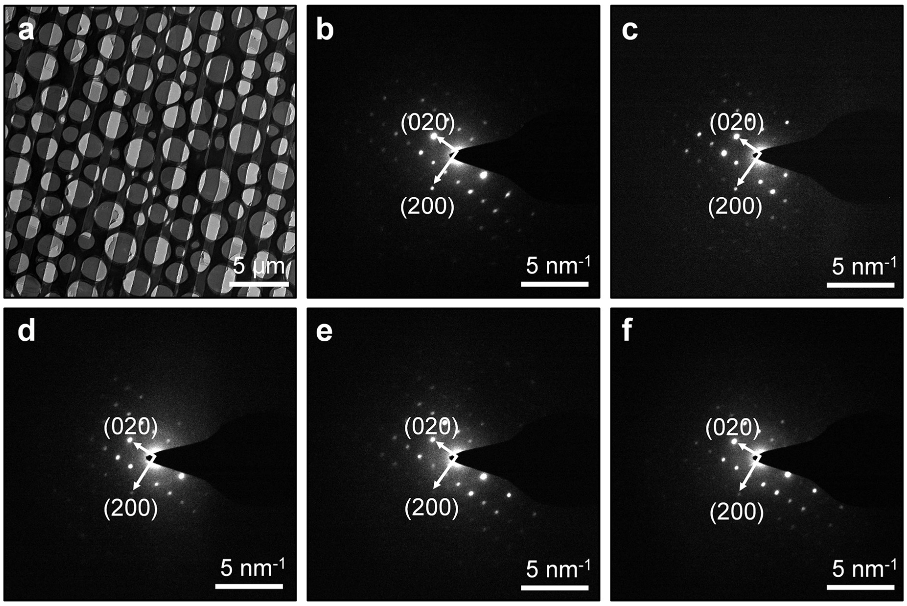


**Supplementary Figure 9 | Characterizations of the C8-BTBT crystal array by TEM. a,** TEM image and **b-f,** the corresponding electron diffraction patterns of the C8-BTBT crystal array.

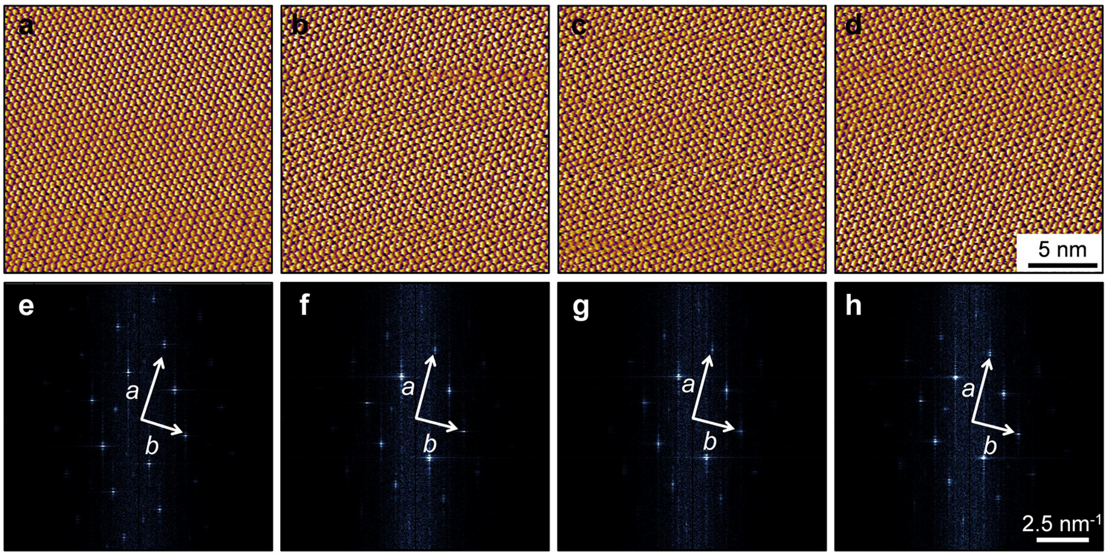


**Supplementary Figure 10 | Characterizations of the C8-BTBT crystal array by AFM. a-d,** High-resolution AFM images and **e-h,** the corresponding FFT patterns of the C8-BTBT crystal array.

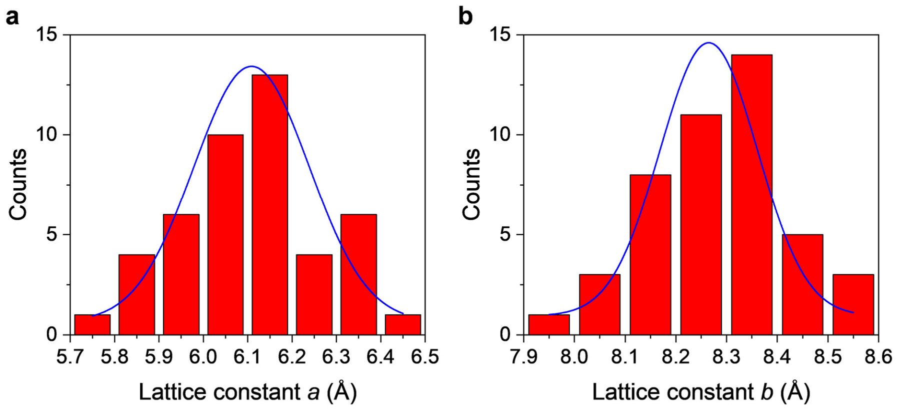


**Supplementary Figure 11 | Lattice constant analyses of C8-BTBT. a and b,** Histograms of lattice constants *a* and *b* acquired from 45 FFT patterns of the C8-BTBT crystal array, respectively.

## IV. Optical anisotropy analyses of C8-BTBT

The optical absorption process in organic semiconductors is related to the excitation of charge carriers from the highest occupied molecular orbital (HOMO) to the lowest unoccupied molecular orbital (LUMO). According to the Davydov theory[11], the anisotropic optical absorption of herringbone-type organic semiconductor crystals is attributed to the orientation difference of adjacent molecules[12,13]. The resonant interaction between two differently oriented adjacent molecules will lead to the Davydov splitting of molecular terms. The Davydov components can be identified by the dimer energies of exciton states:

$$E_{\pm}^{*} = E_0 + E^{*} + D' \pm I_{12} \tag{S9}$$

where $E_0$ and $E^*$ are the energies of ground and excited states, respectively, $D'$ is the Coulomb interaction energy in the excited state, and $I_{12}$ is the resonance interaction energy between the two adjacent molecules. Therefore, the energy exchange between two adjacent molecules will lead to the energy shift and splitting of absorption peaks (Supplementary Fig. 12b). According to the measured absorption peak shift in Fig. 2g, the energy difference caused by polarized absorption can be calculated as:

$$\Delta E = \frac{hc}{\lambda_{90}} - \frac{hc}{\lambda_0} = (6.262\times10^{-34}\ \mathrm{Js})\times(3\times10^{8}\ \mathrm{ms^{-1}})\times\left(\frac{1}{356\times10^{-9}\mathrm{m}} - \frac{1}{360\times10^{-9}\mathrm{m}}\right)$$

$$= 6.02\times10^{-21}\ \mathrm{J} = 0.04\ \mathrm{eV} \tag{S10}$$

where $\lambda_{90}$ and $\lambda_0$ respectively refer to the wavelengths corresponding to 0° and 90° absorption peaks. Meanwhile, the peak intensity can vary greatly in different polarization states. The polarization dependence of the Davydov components can be determined by the transition dipole moments (**μ**) from ground to excited states[13]. In a dimer system (two molecules in a crystal lattice), **μ** can be expressed as:

$$\boldsymbol{\mu}_{\pm} = \frac{1}{\sqrt{2}}[\langle\Psi_1\Psi_2 \,|\, q\mathbf{r} \,|\, \Psi_1\Psi_2^{*}\rangle \pm \langle\Psi_1\Psi_2 \,|\, q\mathbf{r} \,|\, \Psi_1^{*}\Psi_2\rangle] = \frac{1}{\sqrt{2}}(\boldsymbol{\mu}_2 \pm \boldsymbol{\mu}_1) \tag{S11}$$

where $q$ is the elementary charge, $\mathbf{r}$ is the net distance of charge displacement, $\Psi$ and $\Psi^*$ respectively represent the wavefunctions of ground and excited states, and the subscripts 1 and 2 refer to the two adjacent herringbone molecules (namely Molecule 1 and Molecule 2, respectively). The directions of **μ** in each molecule are marked by red arrows in Supplementary Fig. 12a according to the

density function theory-calculated positions in Supplementary Table 4. For the transition from ground to the first excited state, the angle between **μ** and the molecular π plane is ~0.6°. Therefore, the arrangement of the in-plane projections of **μ** can be approximated to be the same as that of the herringbone-stacked C8-BTBT molecules. Consequently, we can label $\boldsymbol{\mu}_1$ and $\boldsymbol{\mu}_2$ respectively in two adjacent C8-BTBT molecules of a crystal lattice (Fig. 2f). By simple geometrical addition of vectors, we obtain the projections of **μ** along *a* axis ($\boldsymbol{\mu}_a = \boldsymbol{\mu}_2+\boldsymbol{\mu}_1$) and along *b* axis ($\boldsymbol{\mu}_b = \boldsymbol{\mu}_2-\boldsymbol{\mu}_1$). Since the absorption intensity can be described by the oscillator strength (*f*), which is proportional to the dipole strength ($D = |\boldsymbol{\mu}|^2$) (ref. [13]). The anisotropic ratio of the peak intensity can be roughly estimated as $1/\tan^2(\alpha/2) = 3.8$ with a herringbone angle $\alpha$ of 54.2°. Note that due to the Davydov shift of the absorption peak, the absorption anisotropic ratio at a fixed wavelength can be larger (*e.g.*, ~5.6 at 365 nm, Supplementary Fig. 13).

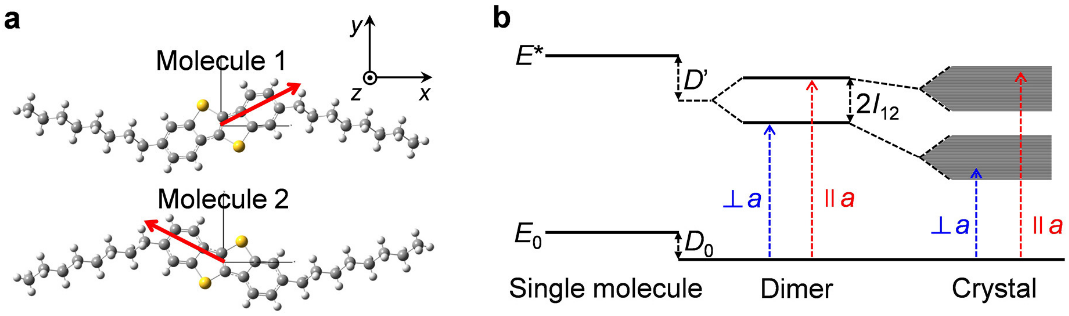


**Supplementary Figure 12 | Analyses of the anisotropic optical absorption of C8-BTBT. a,** Projections of **μ** (marked in red arrows) in molecular π planes of two adjacent herringbone-stacked C8-BTBT molecules. The calculated positions of **μ** are shown in Supplementary Table 4. **b,** Schematic illustrations of Davydov splitting in different systems[11].

**Supplementary Table 4** | Calculated components of **μ** in orthogonal coordinate, with *x* and *y* axes in molecular π plane and *z* axis normal to molecular π plane. The corresponding dipole strength and oscillator strength are also listed.

| Excited state | $\boldsymbol{\mu}_x$ | $\boldsymbol{\mu}_y$ | $\boldsymbol{\mu}_z$ | *D* | *f* |
|---|---|---|---|---|---|
| 1 | ±2.3547 | 1.1661 | -0.0270 | 6.9053 | 0.6199 |
| 2 | ±1.9813 | 0.0321 | 0.2522 | 3.9903 | 0.3865 |
| 3 | ±0.0006 | 0.0000 | 0.0001 | 0.0000 | 0.0000 |

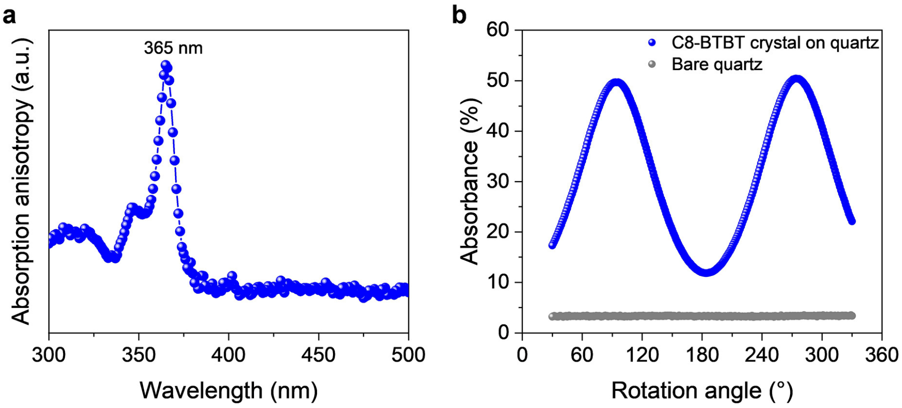


**Supplementary Figure 13 | Anisotropic light absorption of the C8-BTBT crystal on quartz substrate. a,** Light wavelength-dependent absorption anisotropy of the C8-BTBT crystal. The peak is located at 365 nm. **b,** Angle-resolved absorbance of the C8-BTBT crystal under 365 nm polarized light. The absorption anisotropic ratio of C8-BTBT is calculated to be ~5.6 according to $(A_{C8,max}-A_{quartz})/(A_{C8,min}-A_{quartz})$, where $A_{C8,max}$ and $A_{C8,min}$ respectively represent the maximum and minimum absorbance of C8-BTBT, and $A_{quartz}$ is the absorbance of the quartz substrate.

## V. Calculations of the device performance

The hole mobility ($\mu_\mathrm{h}$) of the OPT in the saturation regime is calculated by:

$$\mu_\mathrm{h} = \frac{2L}{WC_\mathrm{i}}\left(\frac{\partial\sqrt{I_\mathrm{DS}}}{\partial V_\mathrm{G}}\right)^2 \quad \text{(S12)}$$

where $L$ and the effective $W$ are respectively 25 μm and 75 μm according to the device structure of the OPT (Supplementary Fig. 14a-c). $C_\mathrm{i}$ is $1.2\times10^{-8}$ F cm$^{-2}$ for a 300 nm-thick $SiO_2$ layer[14], and is ~$1.8\times10^{-8}$ F cm$^{-2}$ in our device configuration with ~200 nm-thick $SiO_2$ serving as the gate dielectric. $\mu_\mathrm{h}$ is calculated to be 2.6 cm$^2$ V$^{-1}$ s$^{-1}$ according to the slope of the black solid line in Supplementary Fig. 14d.

The figure-of-merit parameters of the OPT are calculated as follows. The photosensitivity is defined as the ratio of the photogenerated current ($I_\mathrm{ph}$) to the dark current ($I_\mathrm{dark}$). The responsivity ($R$) of a photodetector is:

$$R = \frac{I_\mathrm{light}\text{-}I_\mathrm{dark}}{P} = \frac{I_\mathrm{ph}}{P} \quad \text{(S13)}$$

where $I_\mathrm{light}$ is the drain current measured under light illumination, and $P$ is the incident light power. The noise equivalent power (NEP) is calculated by:

$$\mathrm{NEP} = \frac{i_\mathrm{n}}{R} \quad \text{(S14)}$$

where $i_\mathrm{n}$ is the noise density in A Hz$^{-0.5}$. The specific detectivity ($D^*$) of a photodetector is extracted according to:

$$D^* = \frac{\sqrt{A\Delta f}}{\mathrm{NEP}} \quad \text{(S15)}$$

where $A$ is the effective device area determined by 75×25 μm$^2$ in the channel region of the OPT, and $\Delta f$ is the signal bandwidth.

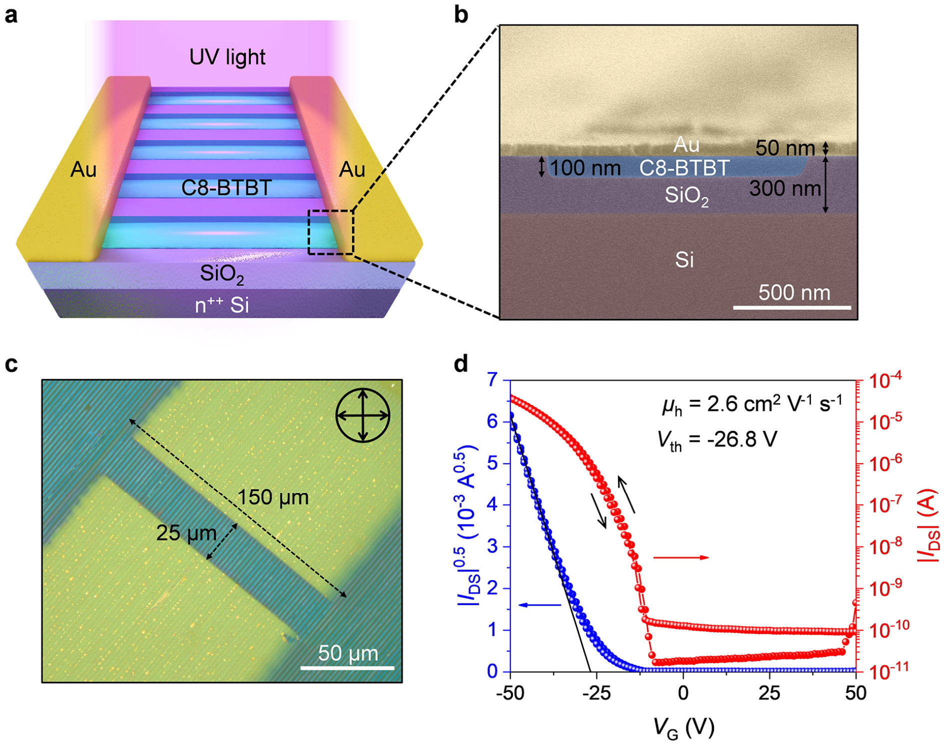


**Supplementary Figure 14 | Device configuration and electrical characterization of the OPT based on C8-BTBT crystal array. a,** Schematic illustration of the bottom-gate top-contact OPT. **b,** Colored cross-sectional SEM image of the OPT, indicating that ~100 nm-thick $SiO_2$ has been etched away and the remaining 200 nm-thick $SiO_2$ underneath the C8-BTBT crystal serves as the gate dielectric. **c,** CPOM image of the OPT. The channel length is 25 μm, while the effective channel width is ~75 μm (half of the measured value) according to the structure of the periodic crystal array. **d,** Double-sweep transfer curves of the OPT in the dark ($V_{DS}$ = -40 V). The black solid line (*i.e.*, tangent line of $\sqrt{I_{DS}}$ versus $V_G$ curve) is used to extract $V_{th}$ and calculate $\mu_h$ in the saturation regime. The negligible hysterisis in the dark is attributed to the high crystal quality and large bandgap of C8-BTBT, which prohibit undesired electron injection in the dark[15].

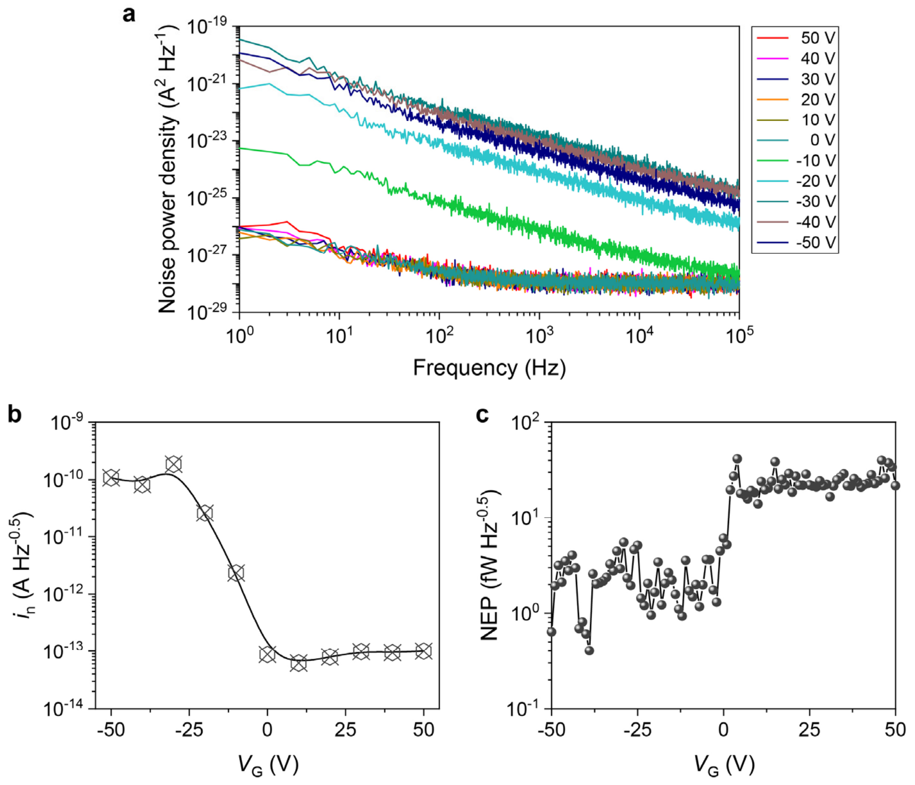


**Supplementary Figure 15 | Noise measurements of the OPT based on C8-BTBT crystal array. a,** Noise power spectral density of the OPT at different $V_G$ ($V_{DS}$ = -40 V). The data was collected by a semiconductor parameter analyzer (Platform Design Automation, Inc. FS-Pro) in an optically shielded probe station. **b,** Dependence of $i_n$ on $V_G$ at a modulation frequency of 1 Hz, showing the low noise that enables ultrasensitive photodetection. **c,** Dependence of NEP on $V_G$ under 365 nm light ($V_{DS}$ = -40 V).

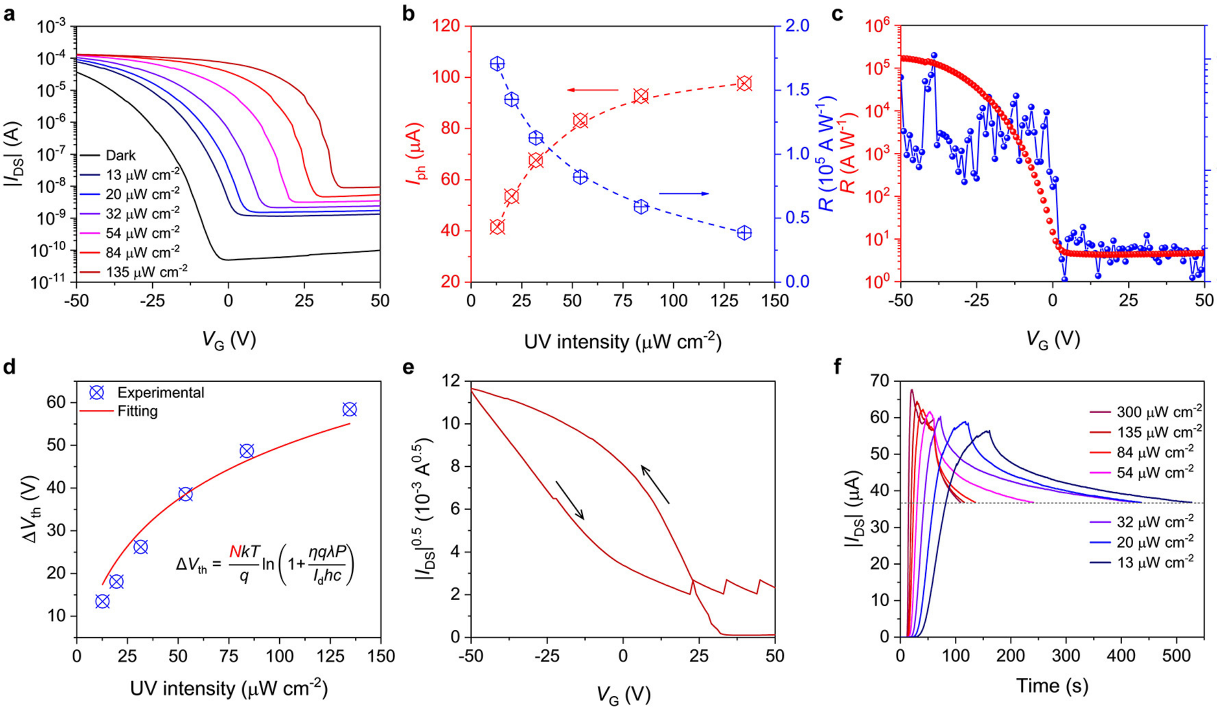


**Supplementary Figure 16 | Photoelectrical chatacterzations of the OPT based on C8-BTBT crystal array. a,** Transfer characteristics of the OPT in the dark and under the illumination of unpolarized UV light with different intensities ($V_{DS}$ = -40 V). **b,** Dependence of $I_{ph}$ and $R$ on UV intensity. **c,** Dependence of $R$ and $D^*$ on $V_G$ (UV intensity = 13 μW cm$^{-2}$). **d,** Dependence of $\Delta V_{th}$ on UV intensity, the empirical parameter $N$ was fitted to be 775.6 with an ideal quantum efficiency $\eta$ of 1. **e,** Double-sweep transfer curve of the OPT under the illumination of unpolarized UV light with an intensity of 135 μW cm$^{-2}$ (scan rate = ~15.5 V s$^{-1}$, $V_{DS}$ = -40 V). **f,** Time-related evolution of $I_{DS}$ upon turning on/off unpolarized UV light with different intensities ($V_{DS}$ = -40 V, $V_G$ = 25 V). The slow decay of $I_{DS}$ after turnning off UV light is due to the gradual detrapping process.

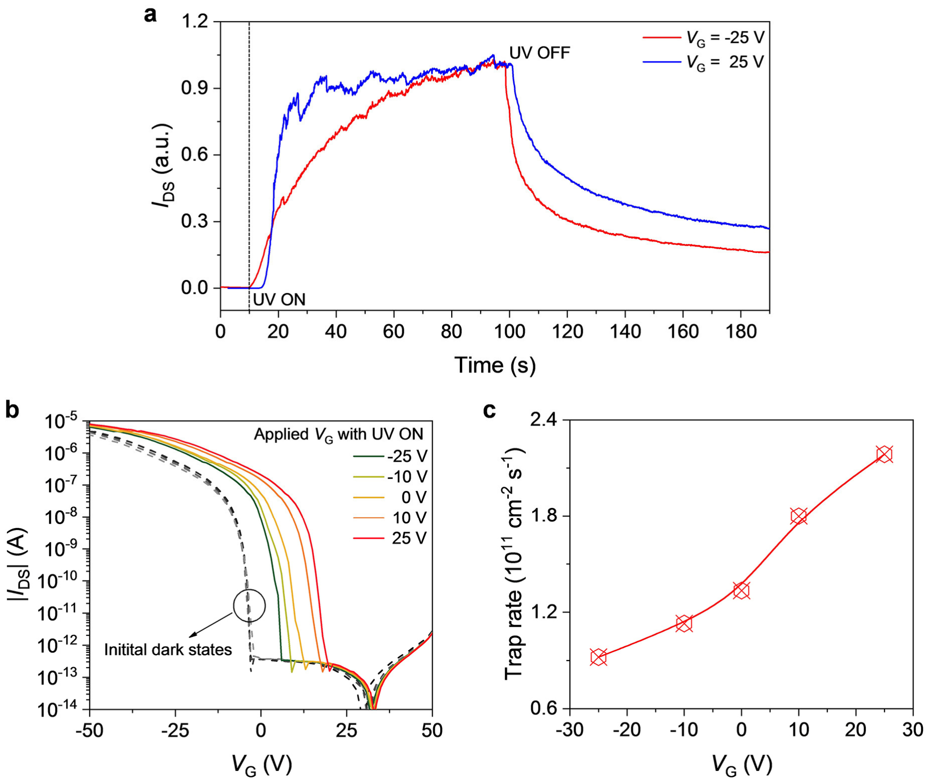


**Supplementary Figure 17 | Factors that influence trap rates of the OPT based on C8-BTBT crystal array. a,** Time-related evolution of $I_{DS}$ upon turning on/off the unpolarized UV light with an intensity of 135 μW cm$^{-2}$ at different $V_G$ ($V_{DS}$ = -40 V). **b,** Transfer characteristics of the OPT measured in the dark after the illumination of 135 μW cm$^{-2}$ unpolarized UV light for 10 s at different $V_G$ ($V_{DS}$ = -40 V). Applying a more positive $V_G$ under illumination helps facilitate electron trapping, leading to a larger threshold voltage shift. **c,** $V_G$-dependent average electron trap rates under 10 s of UV illumination. The average trap rates were estimated according to $\Delta N_{trap}/\Delta t = (C_i \Delta V_{th})/(q\Delta t)$[16], where $\Delta t$ is the illumination time, and $\Delta V_{th}$ is the corresponding threshold voltage shift after 10 s of UV illumination in (b).

## VI. Characterizations of interfacial trap sites on $SiO_2$

To assess the critical role the $SiO_2$/organic semiconductor interface played in charge trapping, bottom-gate top-contact OPTs were fabricated based on different dielectric layers, including bare $SiO_2$, $O_3$-treated $SiO_2$, and $SiO_2$ covered by active group-free divinyltetramethylsiloxane-bis(benzocyclobutene) (BCB) and perfluoro(1-butenyl vinyl ether) polymer (CYTOP) layers. The BCB and CYTOP layers were fabricated by a spin-coating method reported elsewhere[17,18]. 50 nm-thick C8-BTBT film was then thermally deposited on the dielectrics to serve as the active layer, and 50 nm-thick thermally deposited Au electrodes functioned as the source/drain electrodes. The transfer curves of the OPTs based bare $SiO_2$ and $O_3$-treated $SiO_2$ dielectrics exhibit pronounced positive drift under illumination (Supplementary Fig. 18a,b), while the OPTs based on BCB and CYTOP dielectrics exhibit much inferior photoresponse (Supplementary Fig. 18c,d). Since C8-BTBT was thermally deposited simultaneously on the above-mentioned dielectrics to exclude the possible device deviation caused by material growth, and the photoelectric measurements were conducted in a vacuum chamber to exclude the external influence of water/oxygen, we infer that the remaining interfacial active groups on $SiO_2$ surface should be the main source of electron trap sites under illumination.

We further clarify the existence of electron trap sites on $SiO_2$ by scanning kelvin probe force microscopy (SKPM, Asylum Research Cypher S). 50 nm-thick Au electrodes were thermally deposited on different gate dielectrics to function as the source/drain electrodes with heavily n-doped Si ($n^{++}$ Si) serving as the gate. SKPM measurements were conducted by scanning the channel between drain and source electrodes (Supplementary Fig. 19a). During the scan, 10 V bias stress was applied at the gate while the source/drain electrodes were connected to ground, and the time-related potential change between the probe and the scanned surface was recorded. For $SiO_2$ dielectric layer that is susceptible to interfacial active groups such as –OH, a positive gate bias voltage can induce an electrochemical change and lead to the trapping of negative charges (Supplementary Fig. 19b). A possible mechanism can be expressed as $4\text{–OH} + O_2 + 4e^- \rightleftharpoons 4\text{–O}^- + 2H_2O$ in ambient air or $2\text{–OH} + 2e^- \rightleftharpoons 2\text{–O}^- + H_2$ when oxygen is not available[19]. The trapping of negative charges on $SiO_2$ can be evidenced by the rapid potential drop with the increase of scanning time (left of Supplementary Fig. 19c). Further, zero gate bias voltage was applied after scanning the channel with 10 V bias stress for 255 s, a negative potential difference of ~-2.5 V can be observed in the channel region of $SiO_2$ (Supplementary Fig. 20a), indicating the strong capability of negative charge retention on $SiO_2$ surface. In comparison, after

passivating the $SiO_2$ surface by active group-free CYTOP dielectric layer, the surface potential remains quite stable with negligible potential drop with increasing the scanning time (right of Supplementary Fig. 19c), and the potential quickly recovers to 0 V upon applying zero bias stress (Supplementary Fig. 20b), which confirms that the introduction of passivation layer can effectively eliminate the interfacial trap sites.

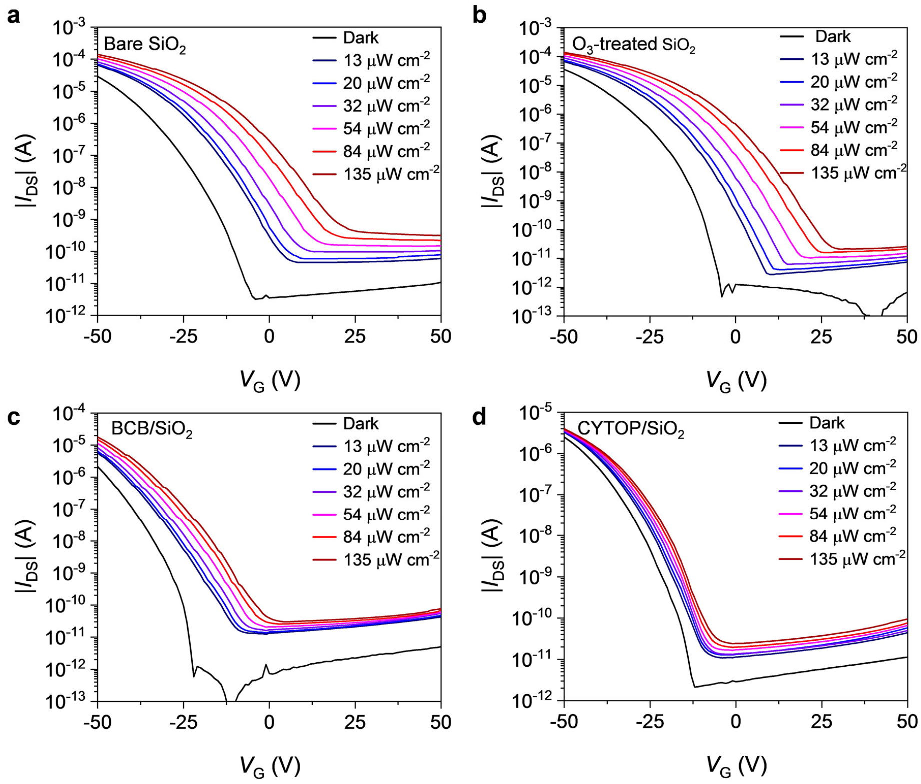


**Supplementary Figure 18 | The influence of surface passivation on photoresponse behavior.** UV light intensity-dependent transfer characteristics of the OPTs based on the same batch of thermally deposited C8-BTBT films on **a,** bare $SiO_2$ dielectrics, **b,** $O_3$-treated $SiO_2$ dielectrics, and active group-free dielectrics including **c,** BCB and **d,** CYTOP ($V_{DS}$ = -40 V).

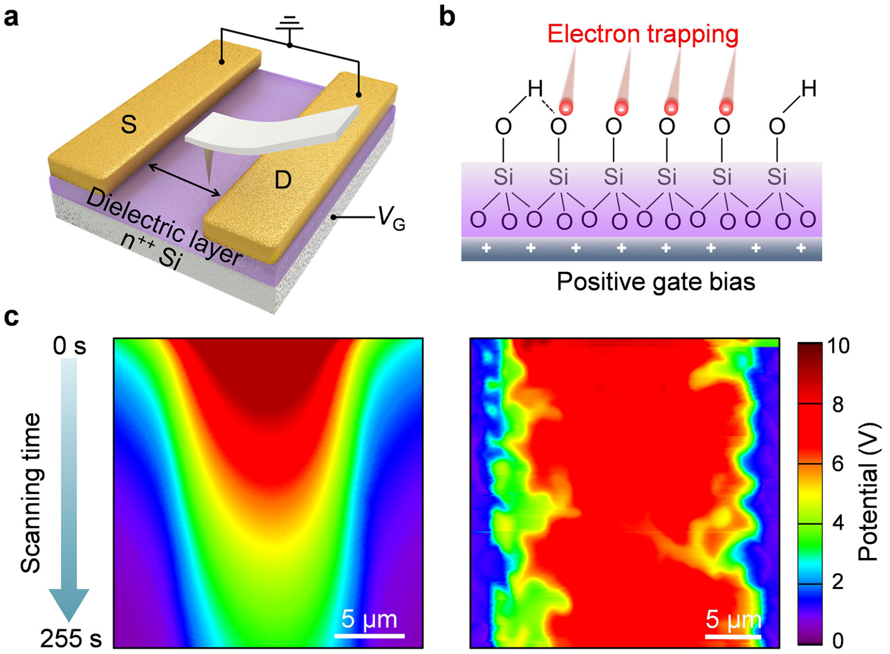


**Supplementary Figure 19 | SKPM measurements for confirming the existence of electron trap sites. a,** Schematic illustration of the device configuration for performing SKPM tests. **b,** Schematic illustration of the electron trapping mechanism on $SiO_2$ dielectrics. **c,** Potential mapping of the channel region on $SiO_2$ dielectrics (left) and CYTOP dielectrics (right) under a continuous gate bias voltage of 10 V.

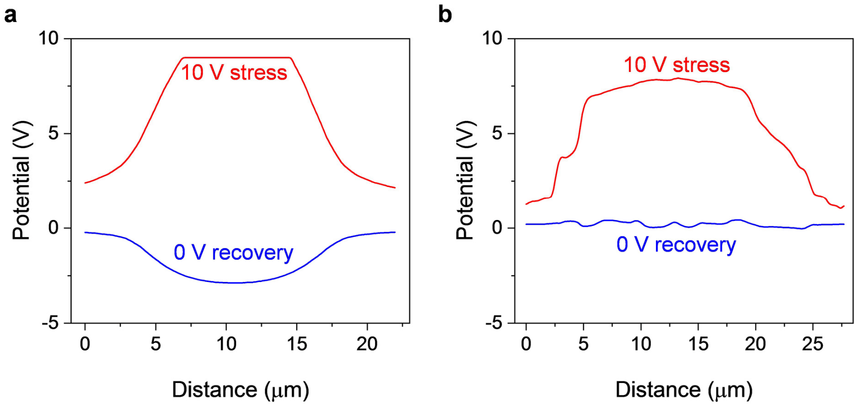


**Supplementary Figure 20 | Comparisons of the potential distributions in the channel regions of different dielectrics.** Potential distributions on **a,** $SiO_2$ dielectrics and **b,** CYTOP dielectrics measured under a gate bias voltage of 10 V (red curves) and after resetting the bias stress to zero (blue curves).

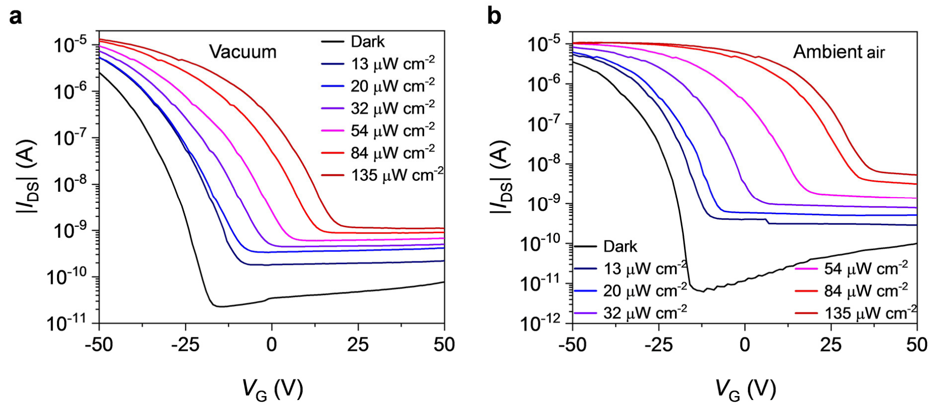


**Supplementary Figure 21 | The influence of testing environment on photoresponse behavior.** UV light intensity-dependent transfer characteristics of the OPT based on C8-BTBT crystal array on $SiO_2$/Si substrate tested in **a,** vacuum and **b,** ambient air ($V_{DS}$ = -40 V). In both cases the transfer curves exhibit pronounced positive drift under light illumination, indicating the strong electron tapping capability of the OPT. Note that the existence of water/oxygen in ambient air can result in the strong p-type doping effect on organic semiconductors[17,20], thus causing a more positive shift of $V_{th}$. However, water/oxygen can also lead to bias stress instability in OPTs (*i.e.*, drift of transfer curves in the dark)[21]. In order to acquire stable and repeatable photoresponse, we conducted all photoelectric measurements in vacuum unless otherwise mentioned.

## VII. Analyses of polarization-dependent photoresponse

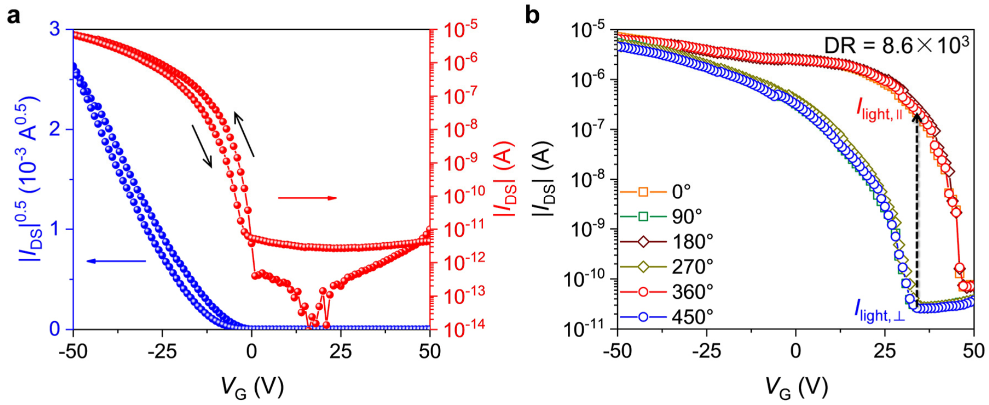


**Supplementary Figure 22 | Stability measurements of the OPT based on C8-BTBT crystal array. a,** Double-sweep transfer curves of the OPT in the dark after preservation in ambient air for over 13 months ($V_{DS}$ = -40 V). **b,** Polarization-dependent transfer curves of the OPT after preservation in ambient air for over 13 months ($V_{DS}$ = -40 V). The slight degradation of $I_{DS}$ is probably caused by water/oxygen adsorption.

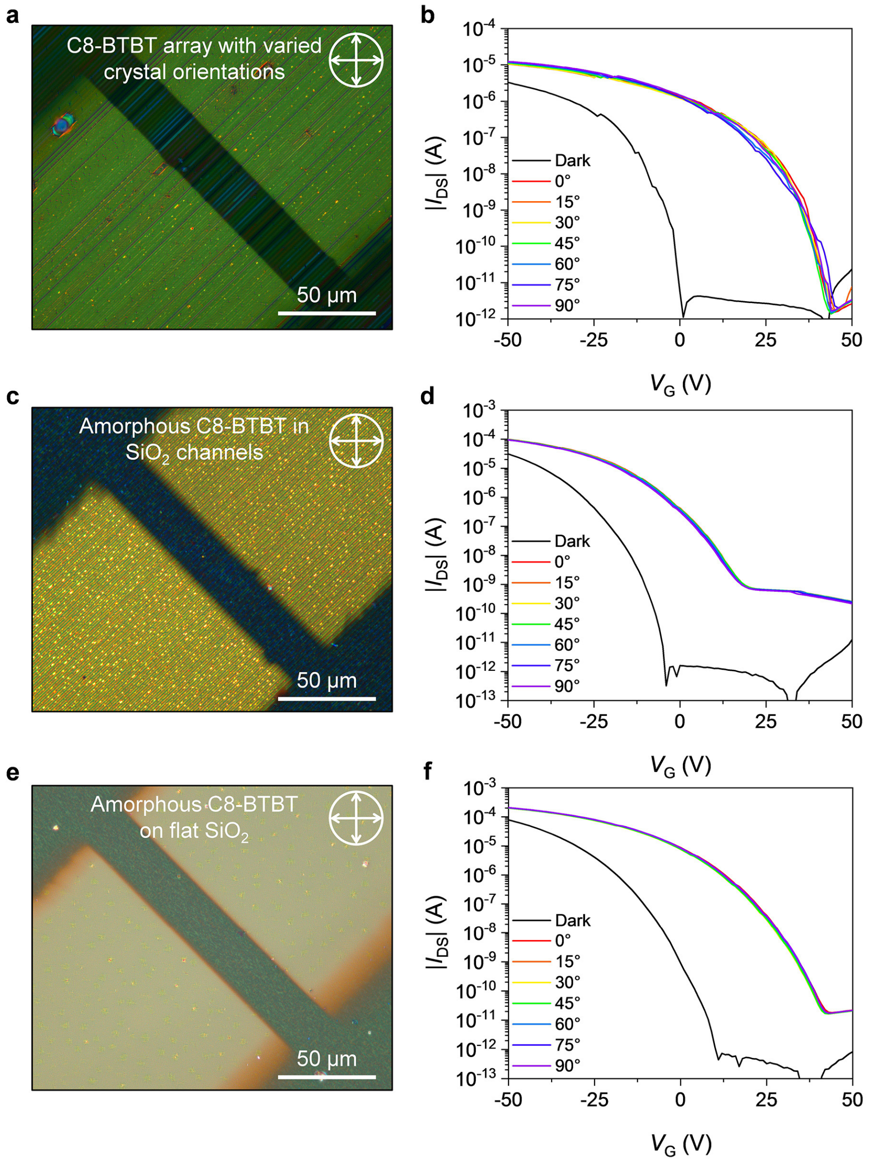


**Supplementary Figure 23 | The influence of active layer crystallinity on polarization sensitivity.** **a,** CPOM image of the OPT based on C8-BTBT array with varied crystal orientations in lyophobic $SiO_2$ micro-channels treated by FTS. **b,** Polarization-dependent transfer curves of the device in (a) ($V_{DS}$ = -40 V). **c,** CPOM image of the OPT based on 50 nm-thick thermally deposited amorphous C8-BTBT in $SiO_2$ micro-channels. **d,** Polarization-dependent transfer curves of the device in (c) ($V_{DS}$ = -40 V). **e,** CPOM image of the OPT based on 50 nm-thick thermally deposited amorphous C8-BTBT on flat $SiO_2$ surface. **f,** Polarization-dependent transfer curves of the device in (e) ($V_{DS}$ = -40 V).

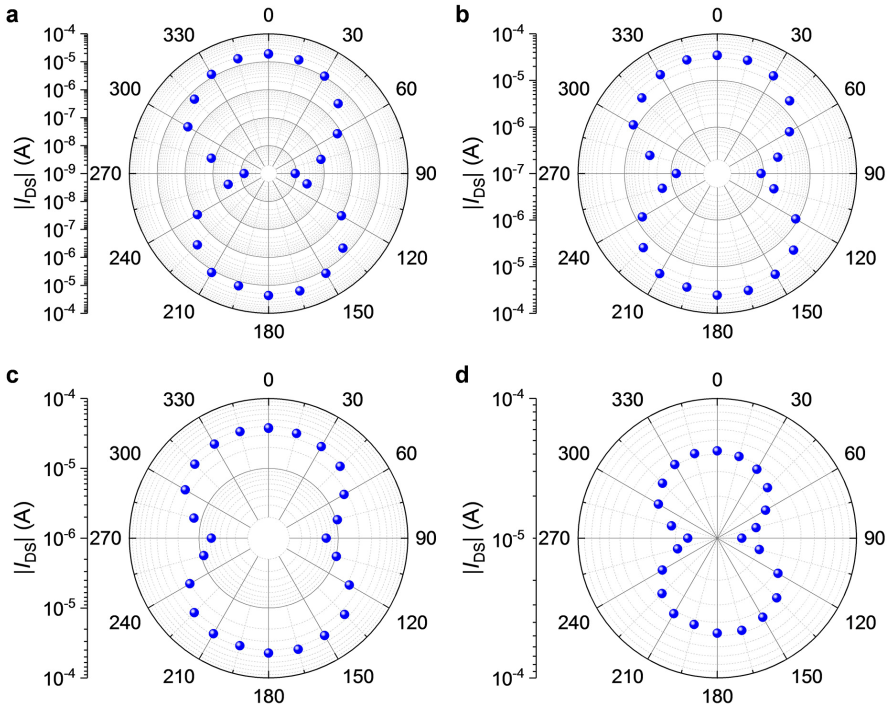


**Supplementary Figure 24 | The influence of applied gate voltage on polarization sensitivity. a-d,** Polarization-dependent variation of $I_{DS}$ at $V_G$ = 20 V, 10 V, 0 V, and -10 V, respectively ($V_{DS}$ = -40 V).

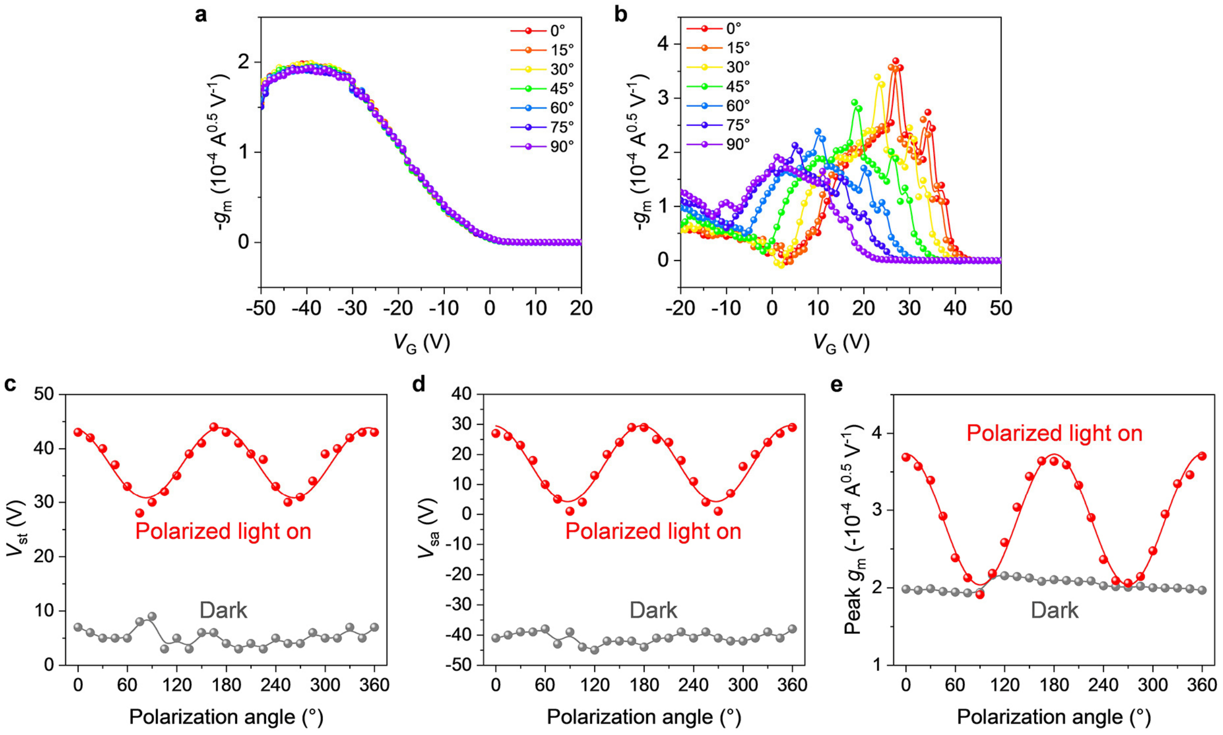


**Supplementary Figure 25 | Analyses of the polarization-dependent transconductance behavior.** **a and b,** Gate voltage-dependent transconductance ($g_m = d\sqrt{I_{DS}}/dV_G$) of the OPT before and under polarized light illumination, respectively. **c-e,** Polarization-dependent $V_{st}$, $V_{sa}$, and peak $g_m$, in the dark (grey curves) and under polarized light illumination (red curves), respectively.

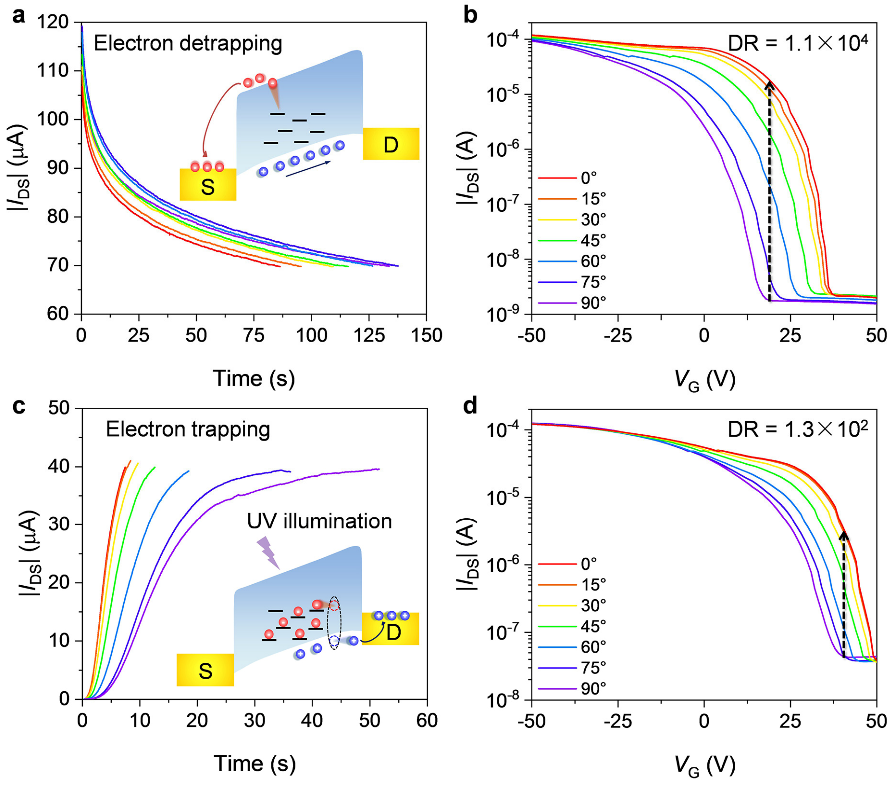


**Supplementary Figure 26 | The influence of available trap sites on polarization sensitivity. a**, Time-related erasing process in the dark to detrap electrons ($V_G$ = -60 V, $V_{DS}$ = -40 V). $I_{DS}$ was reset to the same level to ensure stable and repeatable transfer characteristics in the dark before the measurement of polarized photoresponse (see Supplementary Fig. 28). **b,** Corresponding polarization-dependent transfer curves of the OPT measured after the erasing process in (a). **c,** Time-related programming process under the illumination of polarized UV light to fill trap sites ($V_G$ = 25 V, $V_{DS}$ = -40 V). **d,** Corresponding polarization-dependent transfer curves of the OPT measured after the programming process in (c).

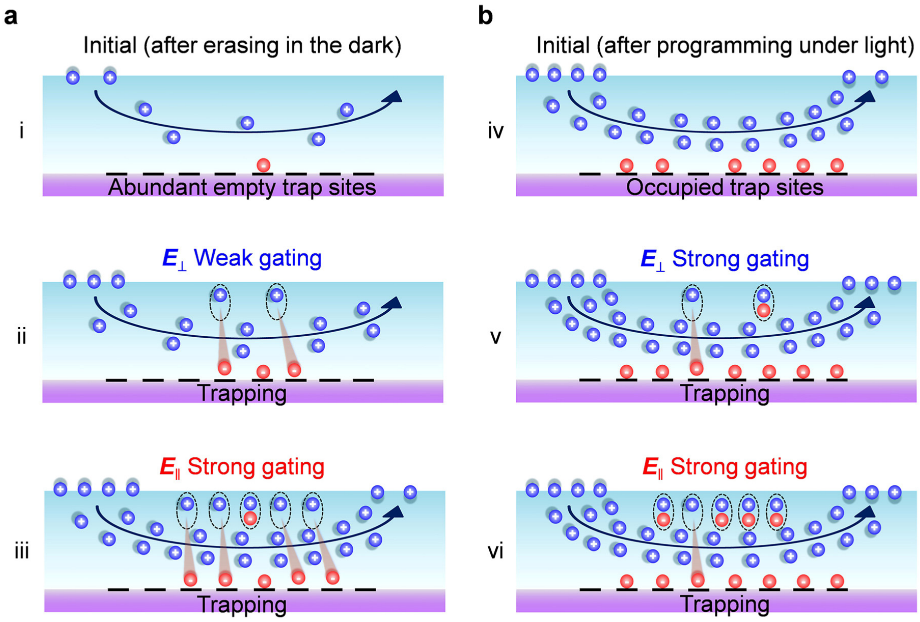


**Supplementary Figure 27 | Explanations on the anisotropic charge trapping mechanism.** Schematic illustrations of the anisotropic charge trapping processes after **a,** erasing in the dark and **b,** programming under light illumination.

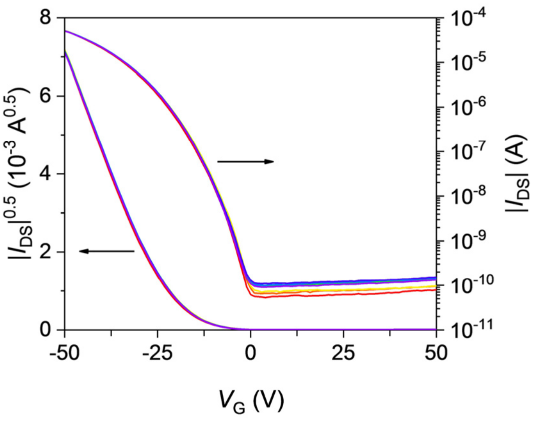


**Supplementary Figure 28 | Dark-state stability of the OPT based on C8-BTBT crystal array.** Transfer curves of the OPT after erasing in the dark ($V_G$ = -60 V, $V_{DS}$ = -40 V), which are highly repeatable with negligible $V_{th}$ shift.

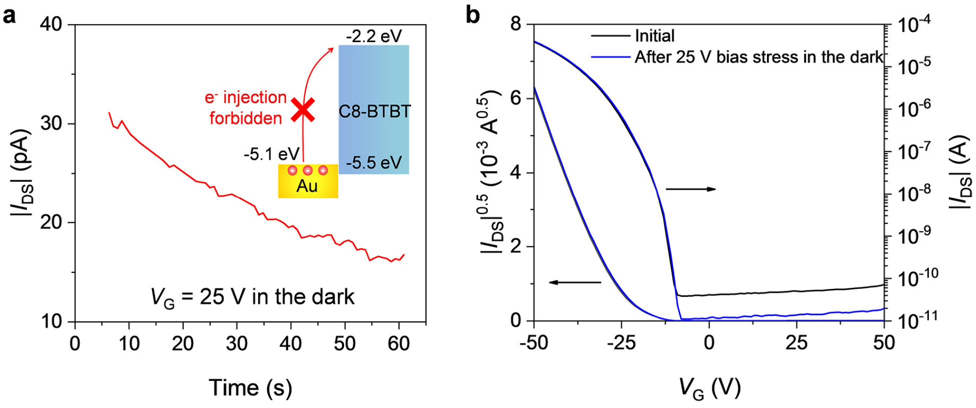


**Supplementary Figure 29 | Analyses of bias stress stability of the OPT based on C8-BTBT crystal array. a,** Time-related programming process in the dark ($V_G$ = 25 V, $V_{DS}$ = -40 V). The inset energy band diagram depicts that no additional electron injection occurs under a continuous gate bias voltage of 25 V in the dark due to the high electron injection barrier. The HOMO level of C8-BTBT was estimated from the onset of oxidation in voltammograms[22], with the LUMO level determined by the difference between the HOMO level (~5.5 eV) and the optical bandgap (~3.3 eV) of C8-BTBT. **b,** Transfer curves of the OPT in the dark before (black curve) and after (blue curve) 25 V gate bias stress in (a), showing negligible $V_{th}$ shift. The minority charge carrier injection phenomenon has been observed in small-bandgap organic semiconductors or those with thermally damaged electrode/active layer interfaces[15]. Injection of electrons from electrodes can lead to positive drift of the transfer curve and increasing $I_{DS}$ under a continuous positive gate bias voltage. Thanks to the relatively large bandgap of C8-BTBT and the careful tuning of Au deposition rate, the OPT exhibits remarkable bias stress stability in the dark.

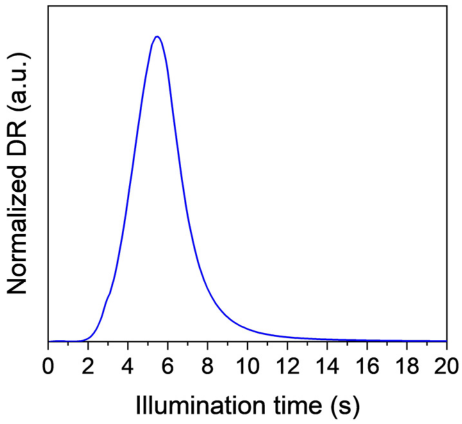


**Supplementary Figure 30 | Dependence of polarization sensitivity on illumination time.** Time-related evolution of DR under continuous illumination of 110 μW $cm^{-2}$ polarized UV light. The peak of DR appears after illuminating the OPT for ~5 s ($V_G$ = 25 V, $V_{DS}$ = -40 V).

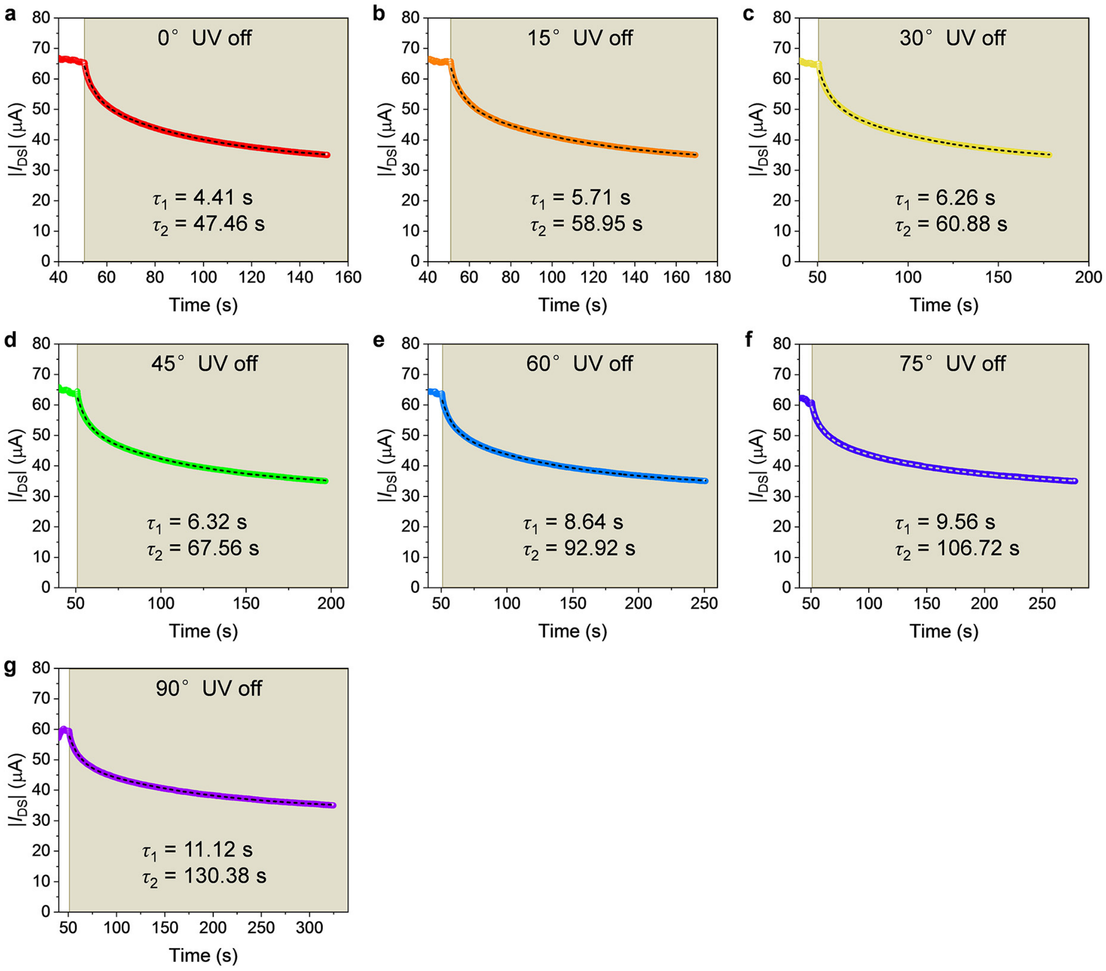


**Supplementary Figure 31 | Dependence of decay curve on polarization angle. a-g,** Enlarged polarization-dependent decay curves of Fig. 3d, showing the persistent photoconductivity upon turning off 110 μW cm$^{-2}$ UV light with polarization angles of 0°, 15°, 30°, 45°, 60°, 75°, and 90°, respectively. The decay curves were fitted by $I_{DS} = I_0 + A_1\exp(-t/\tau_1) + A_2\exp(-t/\tau_2)$ with fitting curves plotted in dashed lines, where $I_0$ is the drain-source current before illumination is removed, $A_1$ and $A_2$ are positive fitting constants, and $\tau_1$ and $\tau_2$ are fast and slow decay time constants, respectively.

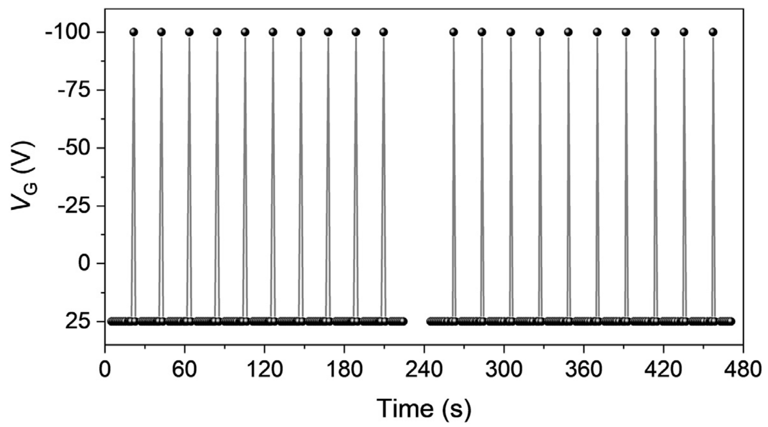


**Supplementary Figure 32 | The applied erasing program for resetting the OPT.** Applied time-dependent $V_G$ corresponding to Fig. 3g ($V_{DS}$ = -40 V). Polarized photoresponse of the OPT was obtained at a continuous $V_G$ of 25 V, and an erasing gate bias voltage ($V_G$ = -100 V) was applied to reset the OPT in the dark. The interval between two erasing $V_G$ was ~22 s.

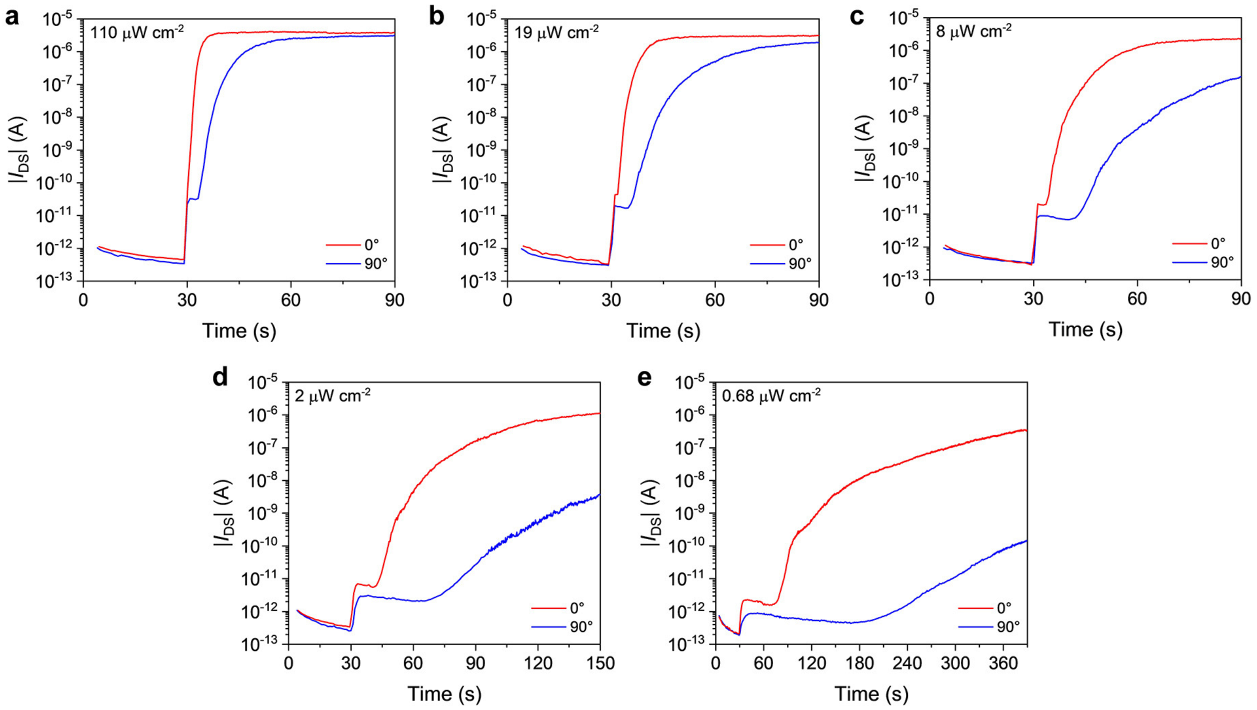


**Supplementary Figure 33 | Dependence of polarized photoresponse behavior on UV intensity. a-e,** Time-dependent polarized photoresponse of the OPT under different UV intensities of 110 μW $cm^{-2}$, 19 μW $cm^{-2}$, 8 μW $cm^{-2}$, 2 μW $cm^{-2}$, and 0.68 μW $cm^{-2}$, respectively ($V_G$ = 25 V, $V_{DS}$ = -40 V). The delay of $I_{DS}$ increase (step-shaped curve) upon illumination is probably caused by the competitive effect between electron-hole recombination and electron trapping under relatively weaker light illumination.

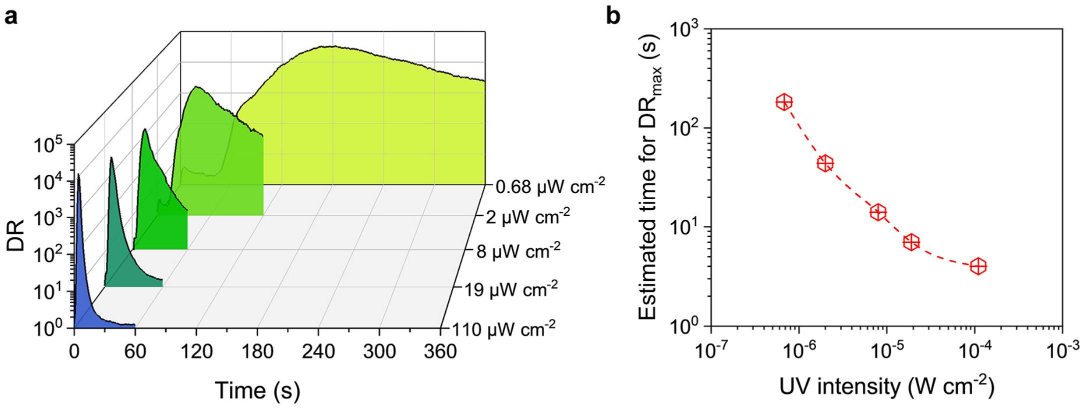


**Supplementary Figure 34 | Dependence of polarization sensitivity on UV intensity. a,** Time-dependent evolution of DR under different UV intensities. **b,** Estimated time for reaching a maximum DR ($DR_{max}$) under different light intensities.

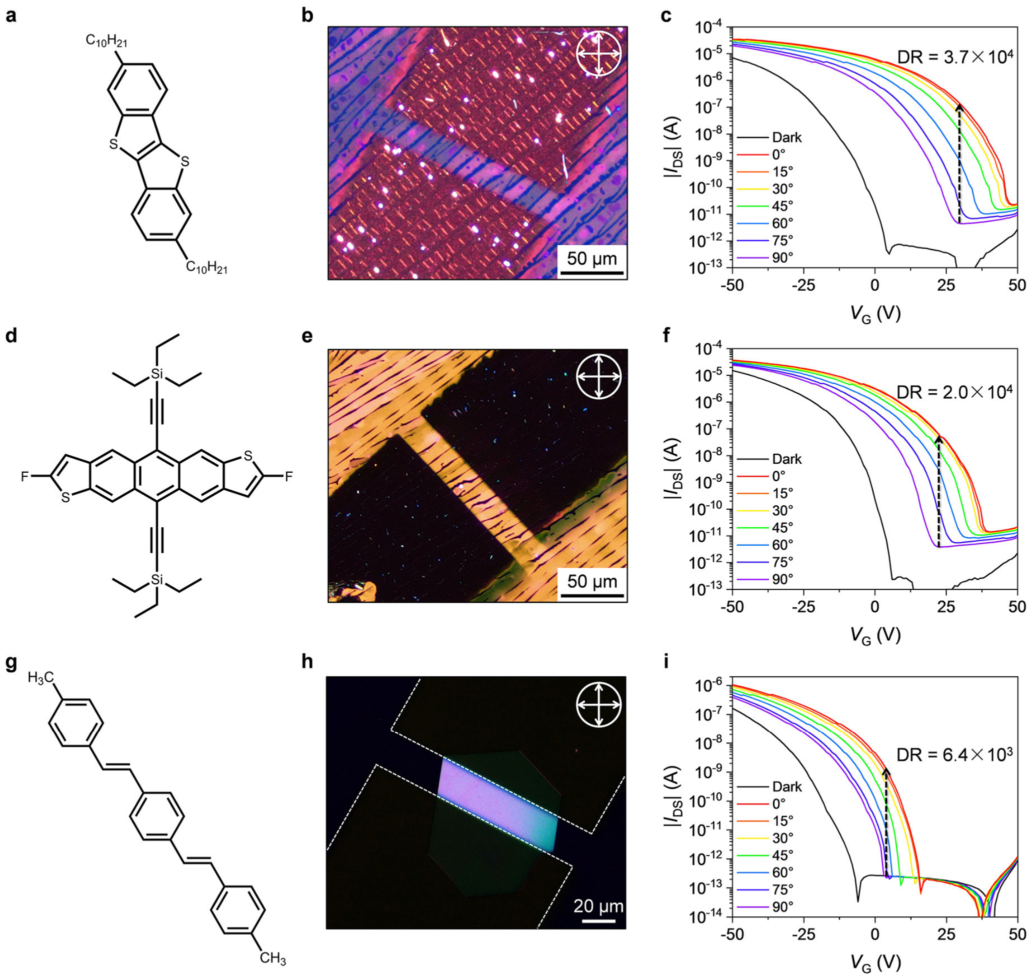


**Supplementary Figure 35 | Universality of the proposed anisotropic charge trapping strategy. a,** Molecular structure of C10-BTBT. **b,** CPOM image of C10-BTBT microribbons on $SiO_2$/Si substrate. **c,** Polarization-dependent transfer curves of the OPT based on C10-BTBT microribbons under 365 nm polarized light with an intensity of 110 μW $cm^{-2}$ ($V_{DS}$ = -40 V). **d,** Molecular structure of dif-TES ADT. **e,** CPOM image of dif-TES ADT microribbons on $SiO_2$/Si substrate. **f,** Polarization-dependent transfer curves of the OPT based on dif-TES ADT microribbons under 525 nm polarized light with an intensity of 11.6 μW $cm^{-2}$ ($V_{DS}$ = -40 V). **g,** Molecular structure of BSB-Me. **h,** CPOM image of a BSB-Me flake on $SiO_2$/Si substrate. **i,** Polarization-dependent transfer curves of the OPT based on a BSB-Me flake under 365 nm polarized light with an intensity of 110 μW $cm^{-2}$ ($V_{DS}$ = -40 V). In all these measurements, the maximum photoresponse direction is set as the 0° reference. Both C10-BTBT and dif-TES ADT microribbons were prepared by a blade-coating method[23], and the narrow-bandgap dif-TES ADT microribbons were decorated with 10 nm wide-bandgap C8-BTBT via thermal evaporation to block undesired electron injection in the dark[15]. The BSB-Me flake was acquired though drop casting the saturated solution on $SiO_2$/Si substrate.

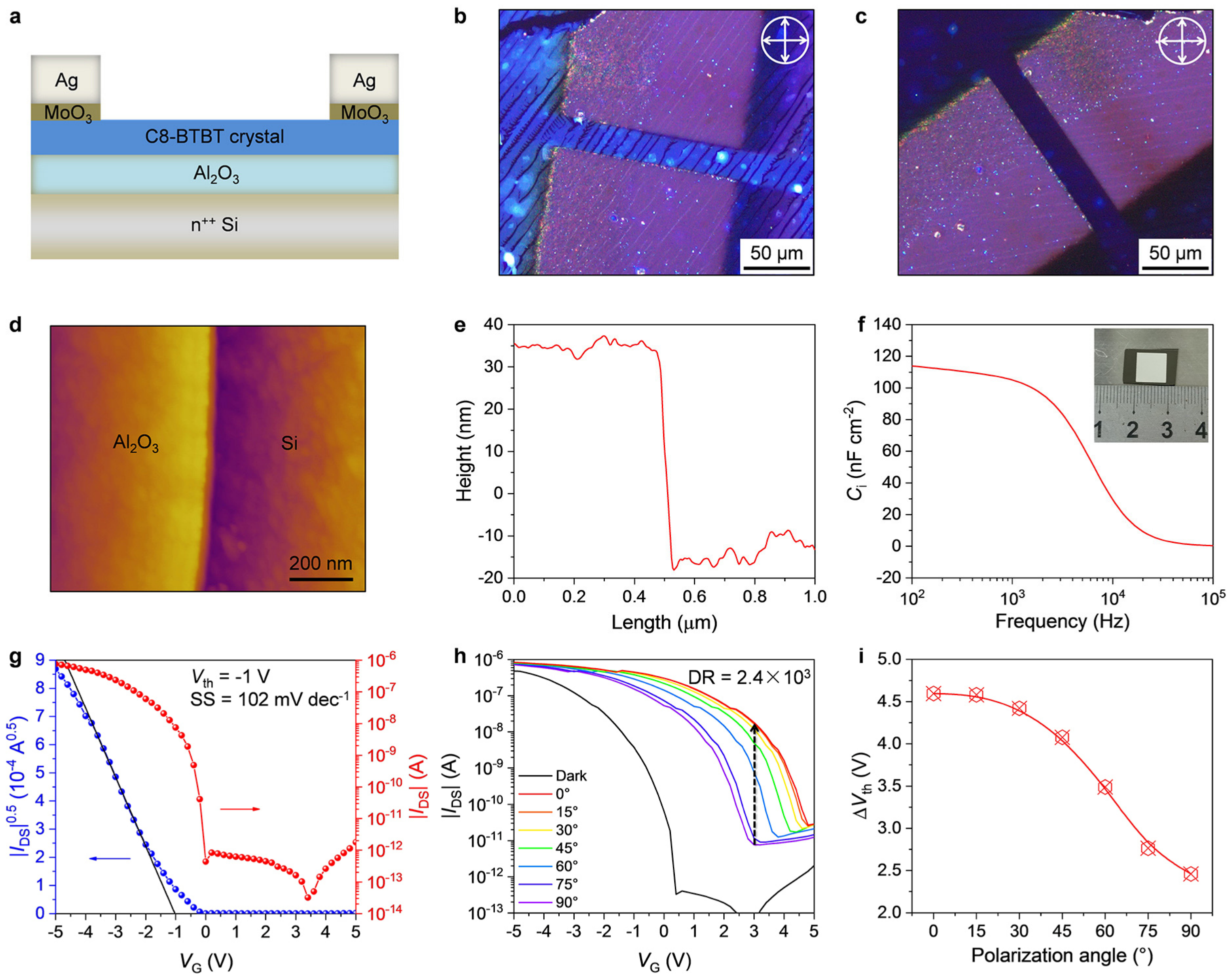


**Supplementary Figure 36 | Demonstration of the polarization-sensitive OPT with low operation voltage. a,** Device structure of the low-operation voltage OPT based on blade-coated C8-BTBT microribbons on $Al_2O_3$. 50 nm $Al_2O_3$ gate dielectric was directly grown on heavily n-doped Si through atomic layer deposition, and 1.5 nm $MoO_3$ layer was thermally evaporated in the electrode contact region to facilitate hole injection and improving device ideality[17]. **b and c,** CPOM images of the low-operation voltage OPT at different sample rotation angles. **d,** AFM characterization and **e,** the corresponding height profile of the $Al_2O_3$/Si boundary, showing ~50 nm thickness of the $Al_2O_3$ gate dielectric layer. **f,** Frequency-dependent capacitance of the $Al_2O_3$ gate dielectric layer at room temperature. The inset is a photograph of the $n^{++}$ Si/$Al_2O_3$/Ag sample with an electrode area of 0.9×0.9 $cm^2$. **g,** Transfer curves of the low-operation voltage OPT in the dark ($V_{DS}$ = -2 V). **h,** Polarization-dependent transfer curves of the low-operation voltage OPT under 365 nm polarized light with an intensity of 110 μW $cm^{-2}$ ($V_{DS}$ = -2 V). **i,** Dependence of $\Delta V_{th}$ on polarization angle of the low-operation voltage OPT, showing a small $\Delta V_{th,PD}$ of ~2.1 V.

**Supplementary Table 5** | Polarization sensitivity comparison of our organic phototransistors with other state-of-the-art polarization-sensitive photodetectors.

| Device composition | $\lambda$ (nm) | DR | Ref. |
|---|---|---|---|
| C8-BTBT crystal array | 365 | ~12,000 | This work |
| C10-BTBT microribbons | 365 | ~37,000 | This work |
| dif-TES ADT microribbons | 525 | ~20,000 | This work |
| BSB-Me flake | 365 | ~6,400 | This work |
| CdSe NWs | 488 | 1.3 | 24 |
| Si NW | 900 | ~2.48 | 25 |
| Carbon nanotubes | 650-1100 | 6.14 | 26 |
| InP NW | 514 | 49 | 27 |
| GeSe flake | 400-950 | 2.16 | 5 |
| $TiS_3$ nanoribbon | 532-808 | 4 | 28 |
| GeAs flake | 400-2000 | 4.4 | 29 |
| $WTe_2$ flake | 514 | 4.9 | 30 |
| $\alpha$-$MoO_3$ crystal | 254 | 5.2 | 31 |
| Te flake | 520-3000 | 8 | 6 |
| $Sb_2Se_3$ nanosheet | 633 | 16 | 32 |
| $MAPbI_3$ NWs | 400-750 | 1.3 | 33 |
| $CsPbBr_3$ NW array | 470 | 2.6 | 8 |
| $(BA)_2PbI_4$ NWs | 492 | 3.62 | 34 |
| Perovskite ferroelectrics | 405 | 15 | 35 |
| $TiS_3$/Si | 405-1050 | 4.6 | 36 |
| BP/$WSe_2$ | 637-1550 | 5.9 | 37 |
| $PdSe_2$/$FA_{1-x}Cs_xPbI_3$ | 200-1550 | 6.04 | 38 |
| BP/plasmonic Au | 1550 | 8.7 | 39 |
| BP/InSe | 455-950 | 10.8 | 40 |
| $PdSe_2$/Si NWs | 200-4600 | 75 | 41 |
| BP/$MoS_2$ | 2500-3500 | ~100 | 42 |
| Graphene/$PdSe_2$/Ge | 265-3043 | 112.2 | 43 |
| BP/ferroelectrics | 1450 | 288 | 44 |
| $Bi_2Se_2S$ integrated with OFET | 532 | 375 | 45 |
| PBnDT-FTAZ:P(NDI2OD-T2) | 350-800 | 1.4 | 46 |
| Pt-complex (1o) | 532 | 2 | 47 |
| DPA microsheet | 450 | 1.9 | 10 |

**Supplementary Table 6** | Comparisons of other figure-of-merit parameters of the state-of-the-art polarization-sensitive photodetectors[a].

| Device composition | *λ* (nm) | *R* (A $W^{-1}$) | *D** (Jones) | $t_r$ | Ref. |
|---|---|---|---|---|---|
| C8-BTBT crystal array | 365 | 1.7×$10^5$ | 1.1×$10^{13}$ (M) | ~4 s @ 300 μW $cm^{-2}$ | This work |
| Si NW | 900 | 2.56×$10^4$ | ~$10^{13}$ (E) | N/A | 25 |
| GeSe flake | 400-950 | 4.25 | N/A | N/A | 5 |
| GeAs flake | 400-2000 | N/A | N/A | 52 ms | 29 |
| α-$MoO_3$ crystal | 254 | 67.9 | N/A | N/A | 31 |
| Te flake | 520-3000 | 1.36×$10^3$ | 1.15×$10^{10}$ (M) | 48.7 μs | 6 |
| $MAPbI_3$ NWs | 400-750 | 4.95 | 2×$10^{13}$ (M) | < 0.1 ms | 33 |
| $CsPbBr_3$ NW array | 470 | 1377 | N/A | 21.5 μs | 8 |
| $TiS_3$/Si | 405-1050 | 3.48×$10^{-2}$ | 1.04×$10^{10}$ (E) | < 20 ms | 36 |
| BP/$WSe_2$ | 637-1550 | ~$10^3$ | ~$10^{14}$ (E) | 0.8 ms | 37 |
| $PdSe_2$/$FA_{1-x}Cs_xPbI_3$ | 200-1550 | 0.31 | ~$10^{13}$ (E) | 3.5 μs | 38 |
| BP/InSe | 455-950 | 1.17×$10^{-2}$ | N/A | 24 ms | 40 |
| $PdSe_2$/Si NWs | 200-4600 | ~0.73 | 3.19×$10^{14}$ (E) | 3.4 μs | 41 |
| BP/$MoS_2$ | 2500-3500 | ~0.9 | 7×$10^9$ (M) | 3.7 μs | 42 |
| Graphene/$PdSe_2$/Ge | 265-3043 | 0.69 | 1.73×$10^{13}$ (M) | 6.4 μs | 43 |
| BP/ferroelectrics | 1450 | 1.06 | 1.27×$10^{11}$ (M) | 0.36 μs | 44 |
| DPA microsheet | 450 | < $10^2$ | ~$10^{12}$ (E) | N/A | 10 |

[a]Note: (M) represents for *D** values that were extracted from measured noise spectra, and (E) represents for *D** values that were estimated based on the dark current.

## VIII. Theoretical estimation of partially polarized light detection

Conventionally, polarization navigation sensors require polarizers integrated with polarization-insensitive photodetectors for the detection of partially polarized skylight. To evaluate their detection ability, we now consider a simplified case where the partially polarized incident light is composed of two orthogonally polarized parts, and the light intensities along these two directions are respectively denoted as $I_{\mathrm{pol},\parallel}$ and $I_{\mathrm{pol},\perp}$. The degree of linear polarization (DoLP) of the incident light is thus $(I_{\mathrm{pol},\parallel}-I_{\mathrm{pol},\perp})/(I_{\mathrm{pol},\parallel}+I_{\mathrm{pol},\perp})$. For a linear polarizer, we define that the light transmittance along its optical axis is $T_\parallel$, and perpendicular to the optical axis is $T_\perp$, its extinction ratio (ER) is thus $T_\parallel/T_\perp$. If the partially polarized light passes through the polarizer (Supplementary Fig. 37a), the finally measured DR by a polarization-insensitive photodetector should be limited by the ratio of transmitted light intensity:

$$\mathrm{DR} \leq (I_{\mathrm{pol},\parallel}T_\parallel + I_{\mathrm{pol},\perp}T_\perp)/(I_{\mathrm{pol},\parallel}T_\perp + I_{\mathrm{pol},\perp}T_\parallel) = \left(\frac{1+\mathrm{DoLP}}{1-\mathrm{DoLP}}\cdot\mathrm{ER}+1\right)\bigg/\left(\frac{1+\mathrm{DoLP}}{1-\mathrm{DoLP}}+\mathrm{ER}\right) \tag{S16}$$

In practical measurements, the DoLP of skylight is only about 60% (or even lower than 30% in UV spectral range)[48], which results in $I_{\mathrm{pol},\parallel}/I_{\mathrm{pol},\perp}$ of only ~4 (or ~1.9 in UV spectral range). Therefore, the increase of ER is favorable for more sensitive detection of partially polarized light (Supplementary Fig. 37b), while a small ER will lead to a degraded DR (Supplementary Fig. 37c) or even indistinguishable signals when DoLP is quite small. Although the emerging anisotropic photoactive materials have provided new opportunities for on-chip polarizer-free polarimeters, their application in partially polarized light detection remains unexplored due to the low polarization sensitivity (DR < 10).

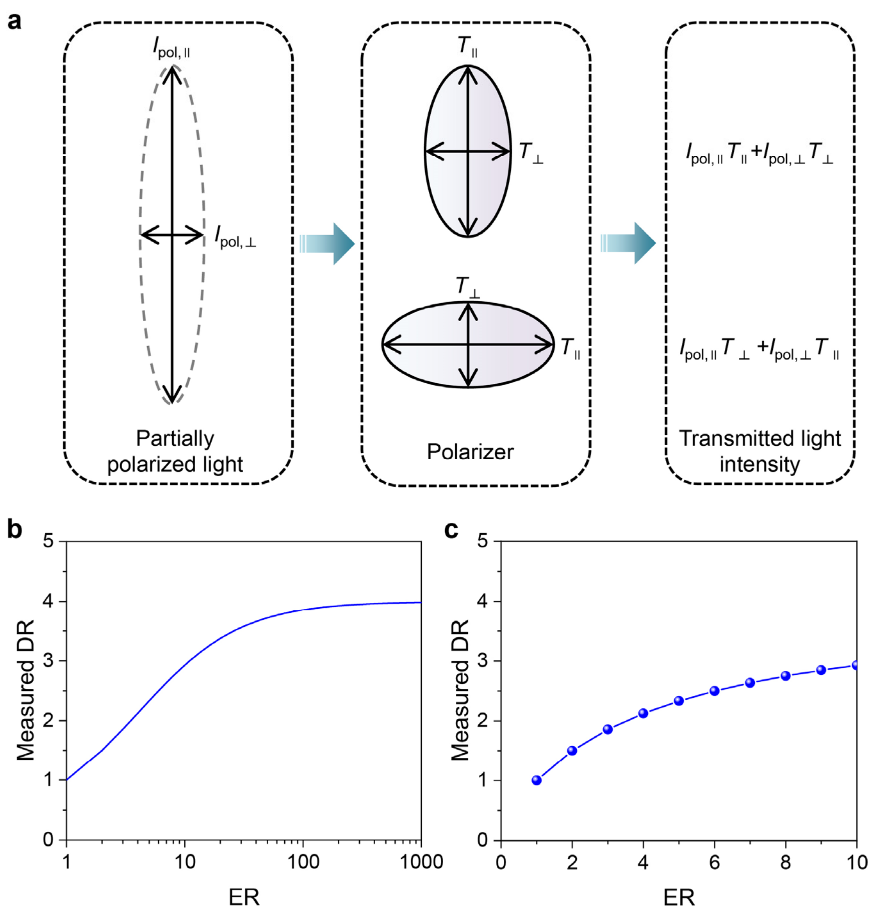


**Supplementary Figure 37 | Estimations of conventional partially polarized light detection by using a polarizer. a,** Schematic illustrations of the detection of partially polarized light. **b,** Predicted relationship between ER of a polarizer and the finally measured DR under a partially polarized light with a DoLP of 60%. **c,** Enlarged plot of (b) with ER in a range of 1-10.

## IX. Polarization navigation measurements

According to the single-scattering Rayleigh model[49], the polarization mode of ideal skylight is symmetrical about the solar meridian (SM), and the e-vector (**E**) of skylight at zenith remains steadily perpendicular to the SM despite the change of time and location, which forms the basis of polarization navigation. By measuring the direction of **E** relative to the heading direction, one can deduce the exact orientation relative to the north. Particularly, although the DoLP in UV spectral region is relatively low, it maintains most reliable under complex weather conditions compared with that of visible light, which makes UV light advantageous for polarization navigation[50].

During polarization navigation measurements, the crystallographic *a* axis of C8-BTBT crystal array was first aligned towards the north direction with the aid of a compass, and this direction was set as the 0° reference direction. Angle-resolved $I_{DS}$ was then collected stepwise by rotating the OPT clockwise in 15° increments and scanning its transfer curve after exposure to skylight for ~10 s. To eliminate the influence of light intensity change caused by the westward movement of the sun for a better fitting, we set two base lines (namely base line 1 and base line 2) according to the valleys and peaks of the sinusoidal curve, respectively (Supplementary Fig. 39a,c). The normalized $I_{DS}$ is obtained after the subtraction of base line 1 by $(I_{DS}\text{-}I_{\text{base line 1}})/(I_{\text{base line 2}}\text{-}I_{\text{base line 1}})$, where $I_{\text{base line 1}}$ and $I_{\text{base line 2}}$ represent the current at base line 1 and base line 2, respectively. (Supplementary Fig. 39b,d). The normalized polarization-dependent $I_{DS}$ is fitted by:

$$I_{DS}(\theta) = I_{\parallel}\cos^2(\theta+\varphi)+I_{\perp}\sin^2(\theta+\varphi) \quad \text{(S17)}$$

where $I_{\parallel}$ and $I_{\perp}$ respectively refer to the drain current along and perpendicular to the strongest photoresponse direction, $\theta$ is the angle with respect to the 0° reference direction, and $\varphi$ is the angle between the 0° reference direction and the strongest photoresponse direction. The fitted strongest photoresponse directions are marked by red arrows in polar coordinates (Supplementary Fig. 40a,c), which are ~-14° and ~-16° relative to the 0° reference direction (compass-pointed north), respectively. We then look for the real-time solar azimuth angle ($\Phi_s$) to determine the theoretical orientations of **E** at zenith. The location of the measurements is in Suzhou Industrial Park, China (120.7°E, 31.3°N), the measuring time period is 3:42-4:17 p.m., 23/09/2021 and 3:27-4:07 p.m., 24/09/2021 corresponding to Fig. 5h,k, respectively. Therefore, $\Phi_s$ is in a range of 252° to 257° and 249° to 255°, respectively. The theoretical orientations of **E** at zenith are thus -18° to -13° and -21° to -15° relative to the north (marked by red arrows in Supplementary Fig. 40b,d). Therefore, both the navigated directions of **E** (~-

14° and ~-16° relative to the compass-pointed north) are located right in the range of the real-time theoretical values, indicating the high accuracy of our simplified celestial compass. More polarization navigation measurements at different times and in different weather conditions are presented in Supplementary Fig. 41 and Supplementary Table 7.

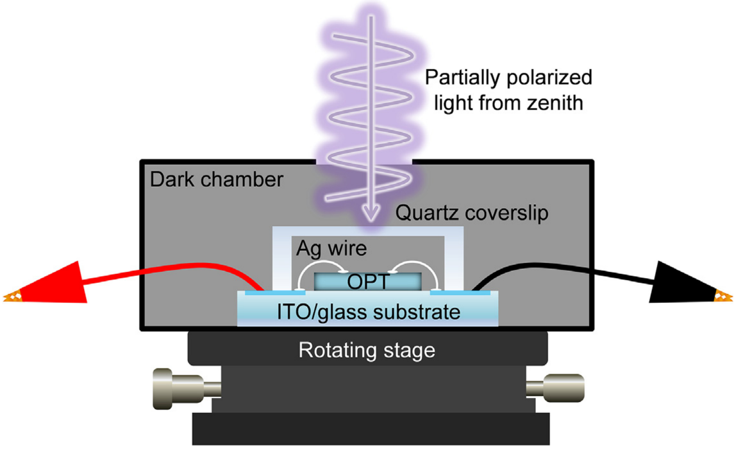


**Supplementary Figure 38 | Experimental setup for outdoor polarization navigation.** Schematic illustration of the experimental setup for the polarization navigation measurements. The OPT was fixed into a dark chamber with a drilled circular window (~1.3 cm in diameter) located right above the device area to receive skylight at zenith and exclude light from the surrounding environment.

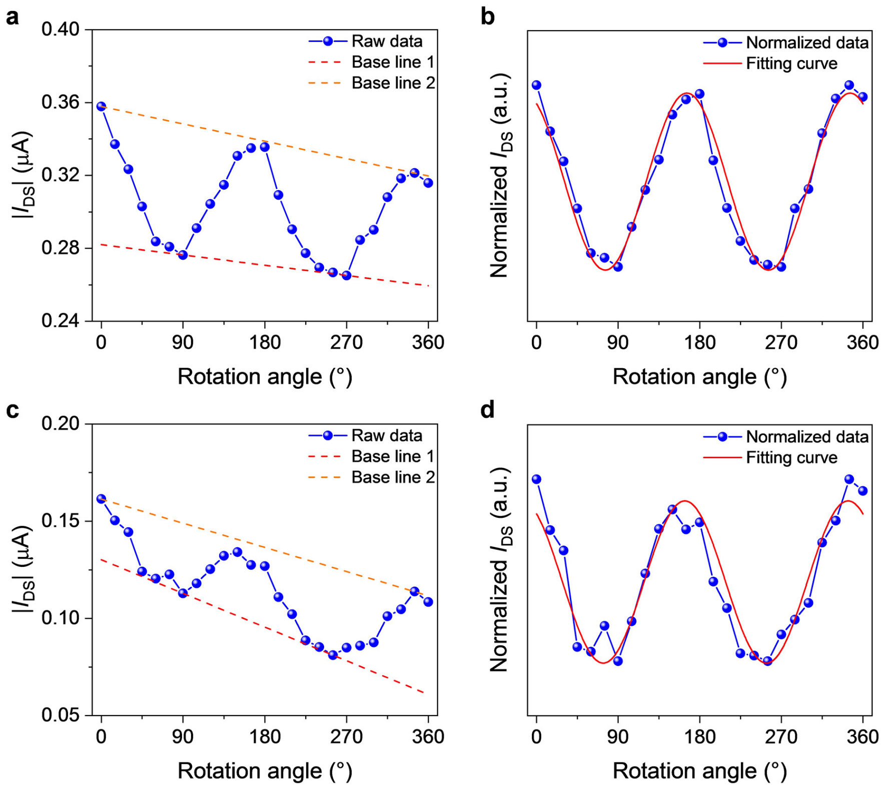


**Supplementary Figure 39 | Data processing for polarization navigation. a,** Polarization-dependent $I_{DS}$ measured under clear sky during a time period of 3:42-4:17 p.m., 23/09/2021 ($V_G$ = -10 V, $V_{DS}$ = -40 V). **b,** Normalized polarization-dependent $I_{DS}$ (blue curve) and the corresponding fitting curve (red curve) after base line subtraction in (a). **c,** Polarization-dependent $I_{DS}$ measured under cloudy sky during a time period of 3:27-4:07 p.m., 24/09/2021 ($V_G$ = -10 V, $V_{DS}$ = -40 V). **d,** Normalized polarization-dependent $I_{DS}$ (blue curve) and the corresponding fitting curve (red curve) after base line subtraction in (c).

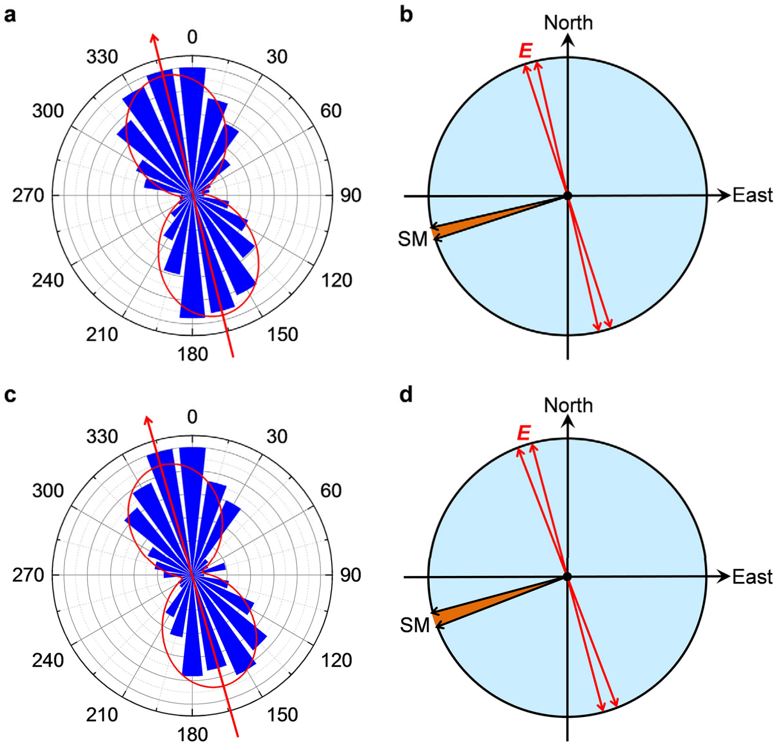


**Supplementary Figure 40 | Comparisons of the navigated polarization directions of skylight with theoretical values. a,** Polar coordinate plot of normalized polarization-dependent $I_{DS}$ measured under clear sky at 3:42-4:17 p.m. (23/09/2021). The angle between the 0° reference direction and the fitted strongest photoresponse direction is ~-14°. **b,** Corresponding real-time orientations of SM and **E** at zenith. $\Phi_s$ is in a range of 252° to 257°, resulting in an **E** direction of -18° to -13° relative to the north direction. **c,** Polar coordinate plot of normalized polarization-dependent $I_{DS}$ measured under cloudy sky at 3:27-4:07 p.m. (24/09/2021). The angle between the 0° reference direction and the fitted strongest photoresponse direction is ~-16°. **d,** Corresponding real-time orientations of SM and **E** at zenith. $\Phi_s$ is in a range of 249° to 255°, resulting in an **E** direction of -21° to -15° relative to the north direction.

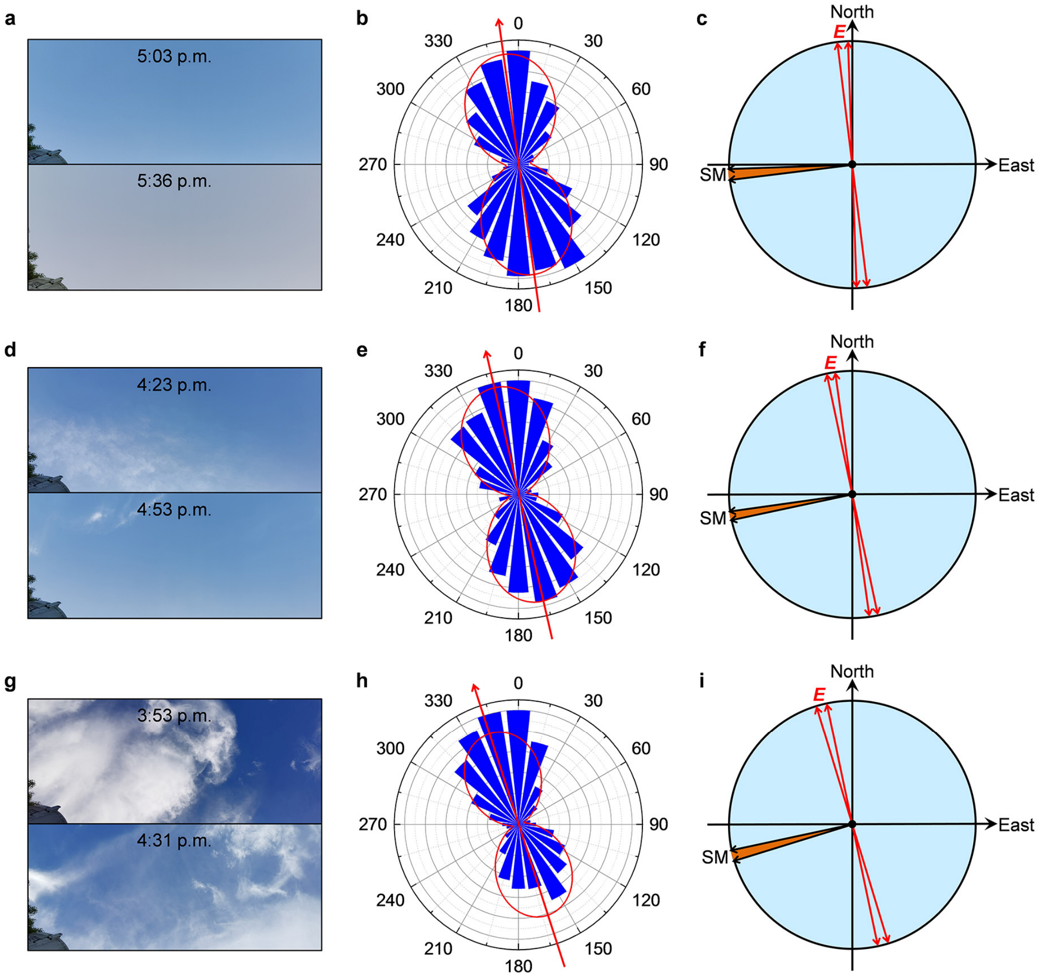


**Supplementary Figure 41 | Polarization navigation results at different times and in different weather conditions. a,** Real-time photograph of the clear sky at 5:03-5:36 p.m. (23/09/2021). **b,** Corresponding polar coordinate plot of normalized polarization-dependent $I_{DS}$. The angle between the 0° reference direction and the fitted strongest photoresponse direction is ~-8°. **c,** Corresponding real-time orientations of SM and **E** at zenith. $\Phi_s$ is in a range of 263° to 268°, resulting in an **E** direction of -7° to -2° relative to the north direction. **d,** Real-time photograph of the cloudy sky at 4:23-4:53 p.m. (24/09/2021). **e,** Corresponding polar coordinate plot of normalized polarization-dependent $I_{DS}$. The angle between the 0° reference direction and the fitted strongest photoresponse direction is ~-13°. **f,** Corresponding real-time orientations of SM and **E** at zenith. $\Phi_s$ is in a range of 258° to 262°, resulting in an **E** direction of -12° to -8° relative to the north direction. **g,** Real-time photograph of the cloudy sky at 3:53-4:31 p.m. (25/09/2021). **h,** Corresponding polar coordinate plot of normalized polarization-dependent $I_{DS}$. The angle between the 0° reference direction and the fitted strongest photoresponse direction is ~-18°. **i,** Corresponding real-time orientations of SM and **E** at zenith. $\Phi_s$ is in a range of 253° to 258°, resulting in an **E** direction of -17° to -12° relative to the north direction.

**Supplementary Table 7** | Summary of polarization navigation measurements conducted at different times and in different weather conditions.

| Weather condition | Time | Date | Real-time $\Phi_s$ | Real-time **E** relative to the north | Navigated **E** relative to the 0° reference |
|---|---|---|---|---|---|
| Cloudy | 3:27-4.07 p.m. | 24/09/2021 | 249° to 255° | -21° to -15° | -16° |
| Clear | 3:42-4:17 p.m. | 23/09/2021 | 252° to 257° | -18° to -13° | -14° |
| Cloudy | 3:53-4:31 p.m. | 25/09/2021 | 253° to 258° | -17° to -12° | -18° |
| Cloudy | 4:23-4:53 p.m. | 24/09/2021 | 258° to 262° | -12° to -8° | -13° |
| Clear | 5:03-5:36 p.m. | 23/09/2021 | 263° to 268° | -7° to -2° | -8° |

## X. Supplementary References


1. Kim, C. H. et al. A compact model for organic field-effect transistors with improved output asymptotic behaviors. *IEEE Trans. Electron Devices* **60**, 1136-1141 (2013).
2. Fayez, M., Morsi, K. M., Sabry, M. N. OTFTs compact models: analysis, comparison, and insights. *IET Circ. Device Syst.* **11**, 409-420 (2017).
3. Baeg, K. J., Binda, M., Natali, D., Caironi, M., Noh, Y. Y. Organic light detectors: photodiodes and phototransistors. *Adv. Mater.* **25**, 4267-4295 (2013).
4. Kim, S. et al. Interstitial Mo-assisted photovoltaic effect in multilayer $MoSe_2$ phototransistors. *Adv. Mater.* **30**, 1705542 (2018).
5. Wang, X. et al. Short-wave near-infrared linear dichroism of two-dimensional germanium selenide. *J. Am. Chem. Soc.* **139**, 14976-14982 (2017).
6. Tong, L. et al. Stable mid-infrared polarization imaging based on quasi-2D tellurium at room temperature. *Nat. Commun.* **11**, 2308 (2020).
7. Feng, X. et al. 2D inorganic bimolecular crystals with strong in-plane anisotropy for second-order nonlinear optics. *Adv. Mater.* **32**, 2003146 (2020).
8. Feng, J. et al. Crystallographically aligned perovskite structures for high-performance polarization-sensitive photodetectors. *Adv. Mater.* **29**, 1605993 (2017).
9. Li, L. et al. Two-dimensional hybrid perovskite-type ferroelectric for highly polarization-sensitive shortwave photodetection. *J. Am. Chem. Soc.* **141**, 2623-2629 (2019).
10. Wang, T. et al. Intrinsic linear dichroism of organic single crystals toward high-performance polarization-sensitive photodetectors. *Adv. Mater.* **34**, 2105665 (2021).
11. Schwoerer, M., Wolf, H. C. *Organic Molecular Solids* Ch. 6 (Wiley-VCH, Weinheim, 2007).
12. Arai, S. et al. Layered-herringbone polymorphs and alkyl-chain ordering in molecular bilayer organic semiconductors. *Adv. Funct. Mater.* **30**, 1906406 (2019).
13. Meyenburg, I. et al. Temperature-resolved optical spectroscopy of pentacene polymorphs: variation of herringbone angles in single-crystals and interface-controlled thin films. *Phys. Chem. Chem. Phys.* **18**, 3825-3831 (2016).
14. Cao, M. et al. Enhanced photoelectrical response of thermodynamically epitaxial organic crystals at the two-dimensional limit. *Nat. Commun.* **10**, 756 (2019).
15. Wu, X. et al. Improving ideality of p-type organic field-effect transistors via preventing undesired minority carrier injection. *Adv. Funct. Mater.* **31**, 2100202 (2021).
16. Podzorov, V., Gershenson, M. E. Photoinduced charge transfer across the interface between organic molecular crystals and polymers. *Phys. Rev. Lett.* **95**, 016602 (2005).
17. Wu, X. et al. Air effect on the ideality of p-type organic field-effect transistors: a double-edged sword. *Adv. Funct. Mater.* **29**, 1906653 (2019).
18. Huang, Y. et al. Effectively modulating thermal activated charge transport in organic semiconductors by precise potential barrier engineering. *Nat. Commun.* **12**, 21 (2021).
19. Un, H. I. et al. Charge-trapping-induced non-ideal behaviors in organic field-effect transistors. *Adv. Mater.* **30**, 1800017 (2018).
20. Jia, R. et al. Unraveling the mechanism of the persistent photoconductivity in organic phototransistors. *Adv. Funct. Mater.* **29**, 1905657 (2019).
21. Iqbal, H. F. et al. Suppressing bias stress degradation in high performance solution processed organic transistors operating in air. *Nat. Commun.* **12**, 2352 (2021).
22. Ebata, H. et al. Highly soluble [1]benzothieno[3,2-b]benzothiophene (BTBT) derivatives for high-

performance, solution-processed organic field-effect transistors. *J. Am. Chem. Soc.* **129**, 15732-15733 (2007).

23. Zhang, X. et al. Fast deposition of an ultrathin, highly crystalline organic semiconductor film for high-performance transistors. *Nanoscale Horiz.* **5**, 1096-1105 (2020).
24. Singh, A. et al. Polarization-sensitive nanowire photodetectors based on solution-synthesized CdSe quantum-wire solids. *Nano Lett.* **7**, 2999-3006 (2007).
25. Das, K., Mukherjee, S., Manna, S., Ray, S. K., Raychaudhuri, A. K. Single Si nanowire (diameter </= 100 nm) based polarization sensitive near-infrared photodetector with ultra-high responsivity. *Nanoscale* **6**, 11232-11239 (2014).
26. Barkelid, M., Steele, G. A., Zwiller, V. Probing optical transitions in individual carbon nanotubes using polarized photocurrent spectroscopy. *Nano Lett.* **12**, 5649-5653 (2012).
27. Wang, J., Gudiksen, M. S., Duan, X., Cui, Y., Lieber, C. M. Highly polarized photoluminescence and photodetection from single indium phosphide nanowires. *Science* **293**, 1455-1457 (2001).
28. Liu, S. et al. Highly polarization sensitive photodetectors based on quasi-1D titanium trisulfide ($TiS_3$). *Nanotechnology* **29**, 184002 (2018).
29. Zhou, Z. et al. Perpendicular optical reversal of the linear dichroism and polarized photodetection in 2D GeAs. *ACS Nano* **12**, 12416-12423 (2018).
30. Zhou, W. et al. Anomalous and polarization-sensitive photoresponse of Td-$WTe_2$ from visible to infrared light. *Adv. Mater.* **31**, 1804629 (2019).
31. Zhong, M. et al. Highly anisotropic solar-blind UV photodetector based on large-size two-dimensional α-$MoO_3$ atomic crystals. *2D Mater.* **5**, 035033 (2018).
32. Song, H. et al. Highly anisotropic $Sb_2Se_3$ nanosheets: gentle exfoliation from the bulk precursors possessing 1D crystal structure. *Adv. Mater.* **29**, 1700441 (2017).
33. Gao, L. et al. Passivated single-crystalline $CH_3NH_3PbI_3$ nanowire photodetector with high detectivity and polarization sensitivity. *Nano Lett.* **16**, 7446-7454 (2016).
34. Ghoshal, D. et al. Catalyst-free and morphology-controlled growth of 2D perovskite nanowires for polarized light detection. *Adv. Opt. Mater.* **7**, 1900039 (2019).
35. Peng, Y. et al. Exploiting the bulk photovoltaic effect in a 2D trilayered hybrid ferroelectric for highly sensitive polarized light detection. *Angew. Chem. Int. Ed.* **59**, 3933-3937 (2020).
36. Niu, Y. et al. Polarization-sensitive and broadband photodetection based on a mixed-dimensionality $TiS_3$/Si p-n junction. *Adv. Opt. Mater.* **6**, 1800351 (2018).
37. Ye, L. et al. Highly polarization sensitive infrared photodetector based on black phosphorus-on-$WSe_2$ photogate vertical heterostructure. *Nano Energy* **37**, 53-60 (2017).
38. Zeng, L. H. et al. Multilayered $PdSe_2$/perovskite Schottky junction for fast, self-powered, polarization-sensitive, broadband photodetectors, and image sensor application. *Adv. Sci.* **6**, 1901134 (2019).
39. Venuthurumilli, P. K., Ye, P. D., Xu, X. Plasmonic resonance enhanced polarization-sensitive photodetection by black phosphorus in near infrared. *ACS Nano* **12**, 4861-4867 (2018).
40. Zhao, S. et al. Highly polarized and fast photoresponse of black phosphorus-InSe vertical p-n heterojunctions. *Adv. Funct. Mater.* **28**, 1802011 (2018).
41. Wu, D. et al. Mixed-dimensional $PdSe_2$/SiNWA heterostructure based photovoltaic detectors for self-driven, broadband photodetection, infrared imaging and humidity sensing. *J. Mater. Chem. A* **8**, 3632-3642 (2020).
42. Bullock, J. et al. Polarization-resolved black phosphorus/molybdenum disulfide mid-wave infrared

photodiodes with high detectivity at room temperature. *Nat. Photon.* **12**, 601-607 (2018).

43. Wu, D. et al. Highly polarization-sensitive, broadband, self-powered photodetector based on graphene/$PdSe_2$/germanium heterojunction. *ACS Nano* **13**, 9907-9917 (2019).
44. Wu, S. et al. Ultra-sensitive polarization-resolved black phosphorus homojunction photodetector defined by ferroelectric domains. *Nat. Commun.* **13**, 3198 (2022).
45. Ran, W. et al. Integrated polarization-sensitive amplification system for digital information transmission. *Nat. Commun.* **12**, 6476 (2021).
46. Sen, P. et al. Panchromatic all-polymer photodetector with tunable polarization sensitivity. *Adv. Opt. Mater.* **7**, 1801346 (2018).
47. Periyanagounder, D. et al. Fast-response, highly air-stable, and water-resistant organic photodetectors based on a single-crystal Pt complex. *Adv. Mater.* **32**, 1904634 (2019).
48. Horváth, G., Wehner, R. Skylight polarization as perceived by desert ants and measured by video polarimetry. *J. Comp. Physiol. A* **184**, 1-7 (1999).
49. Pomozi, I. n., Horváth, G. b., Wehner, R. d. How the clear-sky angle of polarization pattern continues underneath clouds: full-sky measurements and implications for animal orientation. *J. Exp. Biol.* **204**, 2933-2942 (2001).
50. Barta, A., Horvath, G. Why is it advantageous for animals to detect celestial polarization in the ultraviolet? Skylight polarization under clouds and canopies is strongest in the UV. *J. Theor. Biol.* **226**, 429-437 (2004).
